# The MATH-BTB Protein TaMAB2 Accumulates in Ubiquitin-Containing Foci and Interacts With the Translation Initiation Machinery in *Arabidopsis*

**DOI:** 10.3389/fpls.2019.01469

**Published:** 2019-11-22

**Authors:** Nataša Bauer, Andreja Škiljaica, Nenad Malenica, Genadij Razdorov, Marija Klasić, Martina Juranić, Marko Močibob, Stefanie Sprunck, Thomas Dresselhaus, Dunja Leljak Levanić

**Affiliations:** ^1^Division of Molecular Biology, Department of Biology, Faculty of Science, University of Zagreb, Zagreb, Croatia; ^2^Genos Glycoscience Research Laboratory, Zagreb, Croatia; ^3^Division of Biochemistry, Department of Chemistry, Faculty of Science, University of Zagreb, Zagreb, Croatia; ^4^Cell Biology and Plant Biochemistry, University of Regensburg, Regensburg, Germany

**Keywords:** MATH-BTB, translation initiation, eukaryotic translation initiation factor 3, eukaryotic translation initiation factor 4, cytoskeleton, actin11, proteasomal degradation, Triticum aestivum

## Abstract

MATH-BTB proteins are known to act as substrate-specific adaptors of CUL3-based E3 ligases in the ubiquitin proteasome pathway. Their BTB domain binds to CUL3 scaffold proteins and the less conserved MATH domain targets a highly diverse collection of substrate proteins to promote their ubiquitination and subsequent degradation. In plants, a significant expansion of the MATH-BTB family occurred in the grasses. Here, we report analysis of TaMAB2, a MATH-BTB protein transiently expressed at the onset of embryogenesis in wheat. Due to difficulties in studying its role in zygotes and early embryos, we have overexpressed *TaMAB2* in *Arabidopsis* to generate gain-of-function mutants and to elucidate interaction partners and substrates. Overexpression plants showed severe growth defects as well as disorganization of microtubule bundles indicating that TaMAB2 interacts with substrates in *Arabidopsis*. In tobacco BY-2 cells, TaMAB2 showed a microtubule and ubiquitin-associated cytoplasmic localization pattern in form of foci. Its direct interaction with CUL3 suggests functions in targeting specific substrates for ubiquitin-dependent degradation. Although direct interactions with tubulin could not be confimed, tandem affinity purification of TaMAB2 interactors point towards cytoskeletal proteins including tubulin and actin as well as the translation initiation machinery. The idenification of various subunits of eucaryotic translation initiation factors eIF3 and eIF4 as TaMAB2 interactors indicate regulation of translation initiation as a major function during onset of embryogenesis in plants.

## Introduction

MATH-BTB proteins are members of the BTB protein superfamily, encoded by the genomes of almost all eukaryotes thus far studied. The family of MATH-BTB proteins is known for being involved in proteasomal degradation as substrate specific adaptors of E3 ubiquitin ligase complexes—CULLIN3 (CUL3)-based really interesting new gene (RING) E3 ligases or CLR3 (Hershko and Ciechanover, 1998; Pintard et al., 2003; Gingerich et al., 2007). Within the CLR3 complex, MATH and BTB protein domains act as a single-polypeptide bridge between CUL3 and the substrate, in which the BTB domain binds the CUL3 scaffold protein and the MATH domain selectively binds specific target proteins.

Although common for eukaryotes, MATH-BTB proteins are present in disproportionate amounts in different species’ genomes. While the *Arabidopsis* genome contains only six MATH-BTB genes and humans only two genes, which all encode the ancient and highly-conserved core group of MATH-BTB proteins, an expanded and highly divergent group of MATH-BTB proteins was reported for different grass species (Juranić and Dresselhaus, 2014). Similarly, an expanded group of MATH-BTB proteins was found in animals such as *Cenorhabditis elegans* (Stogios et al., 2005). The core clade is comprised of *MATH-BTB* genes detected in all flowering plants studies including the grasses (Juranić and Dresselhaus, 2014). Due to their significant sequence conservation and constitutive expression, it was hypothesized that core-clade genes regulate ancient pathways in plant development and/or physiology (Thomas, 2006). Functional analyses of core-clade plant MATH-BTB proteins revealed their interaction with transcription factors involved in plant stress tolerance (Weber and Hellmann, 2009; Lechner et al., 2011), flowering (Chen et al., 2015), and fatty acid biosynthesis in seeds (Chen et al., 2013; Ma et al., 2013).

Around 95% of grass MATH-BTB proteins belongs to the expanded clade, indicating a possibility of rapid diversification of their physiological substrates (Gingerich et al., 2007) due to grasses’ accelerated evolution (Salse et al., 2009). To date, only one plant MATH-BTB protein of the expanded clade has been functionally described. This is ZmMAB1 protein from maize, encoded by the *ZmMAB1* gene expressed exclusively in the male and female germ lineages as well as in the zygote (Juranić et al., 2012). ZmMAB1 is a plant homologue of MEL-26, a MATH-BTB protein which mediates the activity of CUL3-based E3 ligase and which is required maternally in *C. elegans* for the transition from meiotic to mitotic division and the formation of the mitotic spindle (Pintard et al., 2003). Both MEL-26 and ZmMAB1 target a microtubule severing protein (MEI-1 and katanin subunit p60, respectively) for degradation to ensure long mitotic spindle assembly (Pintard et al., 2003; Xu et al., 2003; Juranić et al., 2012). The expression of MATH-BTB encoding genes of the expanded clade during reproductive development was also reported in wheat. While *TaMAB1* appeared to be egg-cell specific, two further MATH-BTB genes (*TaMAB2* and *TaMAB3*) were described as being expressed during early embryo development. *TaMAB3* showed a very broad expression pattern throughout development, whereas *TaMAB2* appeared especially interesting as it is *de novo* and transiently expressed after fertilization in the zygote and proembryo, and switched off already three days after pollination (Leljak-Levanić et al., 2013).

Because it is very challenging to elucidate TaMAB2 function and substrates in wheat zygotes and proembryos, we have overexpressed *TaMAB2* in *Arabidopsis* to obtain some first insights into its function, activity, and possible substrates. We report about phenotypes observed in transgenic plants overexpressing *TaMAB2*, the observed disorganization of microtubular structures in epidermal cells, the subcellular localization pattern of TaMAB2 during the cell cycle, its interaction with components of the cytoskeleton, with CUL3 and ubiquitin. We also established a tandem affinity purification approach to identify direct interaction partners and its association with larger subcellular complexes.

## Materials and Methods

### Plant Material and Growth Conditions

Seeds of *Arabidopsis thaliana* (L.) Heynh. ecotype Col-0 were sown in a mix of soil (75%) and sand (25%) followed by stratification at 4°C for 2–3 days. After germination, plants were first grown for 2 weeks at short-day conditions (8 h light; 4,500 lx; 22°C) and then transferred to long-day conditions (16 h light; 4,500 lx; 22°C) with 50% relative humidity. Seeds of winter wheat ecotype Florida were grown on cotton wool under standard growth chamber conditions at 26°C with 16 h of supplementary light during the day period and a relative air humidity of 40 to 60%. Tobacco BY-2 cells (*Nicotiana tabacum* cv. bright yellow-2) suspension cells were cultivated in liquid MS medium (Murashige and Skoog, 1962) containing 4.4 g/L of MS-salts (Duchefa), 30 g/L sucrose, 100 mg/L myo-inositol, 1 mg/L thiamin, and 255 mg/L KH2PO4 (pH 5.7) supplemented with 0.2 mg/L 2,4-D, and kept in the dark at 26°C with shaking at 60–70 rpm. For cell suspension establishment, seeds of *A. thaliana* were surface sterilized in a 1% solution of Izosan G (Pliva) and germinated on solid MS medium supplemented with 30 g/L sucrose and 30 mg/L hygromycin. Roots were excised from 2-week old sterile plantlets and transferred on solid MS medium supplemented with 30 g/L sucrose, 0.5 mg/L BAP, 1 mg/L NAA, 1 mg/L IAA, 1 mg/L 2,4 (MS-BY2). After 2 weeks of incubation in the dark at 24°C, 1 g of induced callus was subcultured in 50 ml of liquid MS-BY2 medium. Suspension was grown in the dark at 24°C with gentle agitation (80 rpm). Thirty milliliters of 1-week old suspension was subcultured to 70 ml of fresh medium every week. Cell suspension cultures were harvested by filtration, rinsed with sterile distilled water and used immediately, or snap frozen in liquid nitrogen and stored at −80°C.

### Bacterial and Yeast Strains

*Escherichia coli* strains DH5α (Woodcock et al., 1989), HST04 (StellarTM Competent Cells, Clonetech, #636763), and DE3 (pRIL) were used for cloning and protein overexpression, respectively. For floral dip transformation, *Agrobacterium tumefaciens* strain GV3101 (pMP90) (Koncz and Schell, 1986) was used and electroporated with binary vectors. For yeast-two-hybrid (Y2H) screens, the yeast host Hfc7 [MAT ura3-52 his3-200 ade2-101 lys2-801 trp1-901, leu2-3112 gal4-542 gal 80-538 LYS2::GAL1UAS-G-AL1TATA-HIS3 URA3::GAL417mers(x3)-CyC1TATA-lacZ)] was used. The Hfc7 strain contains reporter genes (HIS3 and lacZ) integrated into the genome (Feilotter et al., 1994).

### Bioinformatics and Phylogenetic Analysis

The *Triticum aestivum* proteome available in Ensembl Plants database was searched using BLASTp with TaMAB2 amino acid sequence as a query. A list of 46 putative wheat MATH-BTB proteins was obtained and non-redundant full-length sequences were aligned with known MATH-BTB proteins from maize (Juranić and Dresselhaus, 2014), rice (Gingerich et al., 2007; Juranić et al., 2012), and *A. thaliana* (Figueroa et al., 2005; Weber et al., 2005; Gingerich et al., 2007) using Clustal Omega v1.2.4 (Sievers et al., 2011). A phylogeny of MATH-BTB sequences was inferred in SeaView v4.6.1 (Gouy et al., 2010) using the maximum likelihood (ML) method with nearest-neighbor interchange (NNI) and supported with the Shimodaira–Hasegawa (SH) approximate likelihood ratio test (aLRT). The tree was drawn using FigTree v1.4.2 (http://tree.bio.ed.ac.uk/).

### Polymerase Chain Reaction Reactions

All PCR reactions were performed in a gradient thermocycler (Applied Biosystems) in a 20 µl reaction volume containing 0.4 U GoTaq® DNA Polymerase (Promega), 4 µl 5X Green GoTaq^?^Reaction Buffer (Promega), 8 pmol of each primer, 1 µl 10 mM deoxyribonucleotide mix, and 100–200 ng of complementary DNA as a template. An initial denaturation step was performed at 95°C for 2 min, followed by 35 cycles of denaturation at 95°C for 30 s, annealing at 55°C for 30 s, extension at 72°C for 1 min/kb, and a final extension step at 72°C for 5 minutes. The reactions were stored at −20°C.

### Generation of Constructs

#### 35Sp::TaMAB2-TAP

The opening reading frame of *TaMAB2* DNA was amplified from the plasmid *Ubip::TaMAB2-GFP* (Leljak-Levanić et al., 2013) using the forward attB1TaB07 and the reverse TaB07attB2a and b primers (**Supplemental Table S1**). The resulting PCR product was cloned into the donor vector pDONR207 by Gateway BP reaction to generate the entry clones pEntr-TaMAB2a and b. The entry clone pEntr-TaMAB2a was used for a gateway LR reaction (Gateway® LR Clonase™ II Enzyme Mix, Invitrogen) with the destination vector pGWB529 to generate the expression vector *35Sp::TaMAB2-TAP::NOSt*.

#### 35Sp::TaMAB2-GFP

A binary vector was constructed from pEntr-TaMAB2a and pB7FWG2.0 (Karimi et al., 2002) by a Gateway LR reaction (see above). This expression vector with C-terminal green fluorescent protein (GFP) fusion to TaMAB2 (*35Sp::TaMAB2-GFP::35St*) was used for floral dip transformation.

#### 35Sp::RFP-TaMAB2

The entry clone pEntr-TaMAB2b was used for a Gateway LR reaction with the destination vectors pH7WGR2.0 (Karimi et al., 2002) to generate the expression vector *35Sp*::mRFP1*-TaMAB2::35St* with an N-terminal mRFP1 fusion to TaMAB2.

#### 35Sp::GFP-TaMAB2

The entry clone pEntr-TaMAB2b was used for Gateway LR reaction with the destination vector pB7WGF2.0 (Karimi et al., 2002) to generate the expression vector *35Sp*::GFP*-TaMAB2::35S*t with GFP fused N-terminal to TaMAB2.

#### 35Sp::2xGFP-MmMBD

A 2,032 bp chimeric gene carrying *sGFP* (S65T green fluorescent protein gene) and the microtubule binding domain (MBD) of the *MAP4* gene (mouse microtubule associated protein 4, GenBank acc. no. M72414) was amplified from genomic DNA of transgenic *Arabidopsis* plants stably expressing GFP-MmMBD (Camilleri et al., 2002); with the primer pair GFP- and MBD-attB2R (**Supplemental Table S1**). The resulting PCR product was cloned into the donor vector pDONR207 by a Gateway BP reaction and subsequently into the destination vector pB7WGF2.0 by an LR reaction generating the expression vector *35Sp::eGFP-sGFP-MBD::35S*t.

### Transient Transformation and Generation of Transgenic Plants

For transient transformation, the particle gun model PDS100/He (Bio-Rad), 1,100-psi rupture disks, and a vacuum of 28 mm Hg with 6-cm target distance were used for bombardment of tobacco BY-2 cells. A thin and uniform layer of cells in log phase of growth was spread onto a solid MS medium with 200 µg/l 2,4-D and incubated at 26°C for 1–2 h before biolistic transformation with *35S::RFP-TaMAB2* and microtubule labeling construct *35Sp::2xGFP-MmMBD*. After transformation, plates were incubated overnight at 26°C in the dark. Cells were transferred into fresh liquid MS medium supplemented with 2,4-D and cultivated in 35-mm Petri dishes in the dark with continuous shaking. For microscopy, 100 µl of suspension culture was transferred onto cover slips fixed to metal slides provided with an opening (Ø 20 mm) in the center. Experiment was repeated three times. Each time 100 labeled cells were analyzed.

*Arabidopsis* plants (ecotype Col-0) were transformed by the floral dip method (Clough and Bent, 1998) using the *A. tumefaciens* strain GV3101 (pMP90RK). For selection, seeds were surface sterilized with 1% Izosan G (Pliva) and 0.01% (v/v) mucasol for 10 min, cold treated at 4°C for 2 d, and then plated on MS medium with 0.8% (w/v) agar and 2% (w/v) sucrose (germination plates). Transgenic seeds transformed with *35S::2xGFP-MmMBD* were selected by spraying three times with 200 mg/L BASTA (Bayer Crop Science) supplemented with 0.1% (v/v) Tween 3 days after germination. Transgenic seeds transformed with *35Sp::TaMAB2-GFP::35St* and *35Sp::TaMAB2-TAP::NOSt* constructs were selected using 20 mg/L glufosinate-ammonium and 30 mg/L hygromycin, respectively. Plates were incubated in 16-h-light/8-h-dark cycles at 24°C. From each transformation event we selected 10 T1 transgenic lines and confirmed the presence of transgenes by PCR. T1 plants were allowed to self-pollinate to produce T2 and T3 seeds. Three independent *35S::MAB2-GFP* lines (designated further as 80, 81, 82) and six *35S::MAB2-TAP* lines (further designated as EM4, H3, H4, H5, H6, H7) were established. Expression of the fusion TaMAB2–GFP and TaMAB2-TAP proteins in transgenic lines was verified by immunoblotting using anti-GFP (Roche) and peroxidase anti-peroxidase (PAP; Sigma-Aldrich) antibody soluble complex, respectively. PAP antibody soluble complex detects Protein A within TAP tag. For phenotype analysis all six TAP-tagged independent homozygous lines (T3 generation) were used in three biological replicates and an average was calculated for growth parameter characterization. The lines EM4 and H4 were selected for further experiments.

For epidermal cell measurements and visualization of microtubule organization in the *TaMAB2* overexpressing background, we crossed independent homozygous *35S::MAB2-TAP* lines EM4 and H4 (female parent) with *Arabidopsis* microtubule (MT) marker line stably expressing GFP-MmMBD (Marc et al., 1998) generated as described in Camilleri et al. (2002) (male pa-rent, further designated as MAP4). The resulting GFP-labeled F1 progeny (designated as TaMAB2×MAP4 or, specifically EM4×MAP4 and H4×MAP4) was used for confocal laser scanning microscopy (CLSM). Roots of 5-day old seedlings were incubated for 1 min in propidium iodide (PI) solution and rinsed in tap water for another minute. The working solution of PI was prepared from a stock (1 mg/ml in dH_2_O) diluted 1:20 in tap water just before usage. Analysis was performed for both crossbreeds in three biological replicates with six individual seedlings in each.

### Epidermal Cell Measurements

Seeds of EM4, H4, and hybrid lines EM4×MAP4 and H4×MAP4 were plated on germination plates and grown in standard conditions as described above. The *Arabidopsis* MT marker line MAP4 was used as a control. Root lengths and epidermal cell dimensions of 5-day old seedlings were measured using ImageJ v.1.49 (Schneider et al., 2012). For cotyledon epidermal cells, measurements were performed on 50 cells in three biological replicates each including three individual seedlings for each EM4×MAP4 and H4×MAP4 line. For root epidermal cells, measurements were performed on five cells in root hair initiation zone of 15–20 individual seedlings of EM4, H4, and hybrid EM4×MAP4 and H4×MAP4 lines. The measurement and analysis was performed in three biological replicates. Statistical significance of differences between means of wild type and transgenic lines were analyzed by two-tailed T-test with the p value of <0.05 regarded as significant.

### Yeast-Two-Hybrid Assay

Gene-specific primers were designed using Clonetech In-Fusion Primer Design Tool for cloning into *Bam*HI restriction sites. Primer sequences used for cloning are listed in **Supplemental Table S1**.

Each gene of interest was amplified using the 1X In-Fusion CloneAmp™ HiFi PCR Premix (Clontech). Plasmid constructs coding for proteins of interest were N-terminally fused with either the activation domain (AD) or DNA-binding domain (BD) of the Gal4 transcription factor. For Y2H assays, the yeast strain Hfc7 was co-transformed with plasmid constructs of TaMAB2 and its potential interactor in both orientations using a standard lithium-acetate (LiAc) technique (Agatep et al., 1988). Transformants were selected on solid dropout medium lacking leucine and tryptophan. For a histidine prototrophy assay, individual colonies were grown on selective dropout medium lacking leucine, tryptophan, and histidine. This medium contained 13 mM 3-amino-1,2,4-triazole (3-AT) for elimination of non-specific protein-protein interactions. The β-galactosidase assay using X-gal as substrate was performed according to the *Yeast Protocols Handbook* (Clontech). For each experiment six individual colonies were used.

### Duolink *In Situ* Proximity Ligation Assay

Ubiquitination of GFP-tagged TaMAB2 in transgenic protoplasts was tested using a Duolink *In Situ* proximity ligation assay (PLA) assay (OLINK Bioscience, Uppsala, Sweden). The primary antibodies were mouse monoclonal anti-GFP (1:400, 11814460001, Roche) and rabbit polyclonal anti-ubiquitin antibody (1:2,000, AB1690, Chemicon International). Negative controls were performed using *A. thaliana* Col-0 wild type protoplasts, in which the anti-GFP antibody should have no targets.

Protoplasts were isolated from 2-week old seedlings (Zhai et al., 2009) of *A. thaliana* overexpressing TaMAB2-GFP (lines 80 and 82) in Col-0 genetic background. Protoplasts were fixed with 4% paraformaldehyde and adhered to positively charged silane-coated slides. After rehydration in phosphate-buffered saline, protoplasts were blocked for 30 min using Duolink PLA Blocking Solution and incubated in Duolink PLA Antibody Diluent (OLINK Bioscience) containing primary antibodies. Primary antibody incubation lasted for 2 h at room temperature followed by overnight incubation at 4°C. Ubiquitination of GFP-tagged protein was detected using Duolink *In Situ* PLA probes and Duolink *In Situ* Detection Reagents Red according to manufacturer’s instructions (OLINK Bioscience). A minimum of 30 protoplasts emitting a PLA signal was analyzed for both lines in three biological replicates.

### Microscopy

Bright field and fluorescent specimens were observed under a Zeiss Axiovert 200 M. Filter set 09 (450–490 nm excitation, LP 515 nm) was used for GFP fluorescence. Filter set 14 (510–560 nm excitation, 590 nm emission) was used for detection of mRFP1 fluorescence. For Duolink analysis red signals (TX Red) were detected using filter set 31 (BP 565/30 nm excitation, BP 620/60 emission) and blue signals (UV) detected using filter set 49 (G365 nm excitation, BP 445/50 nm emission).

Samples were excited with UV-light produced by a HBO 50/Ac lamp and images were taken with a Nikon DS-5Mc camera. The software EclipseNet plug (Nikon) was used to measure and merge fluorescence images. CLSM was performed using either the confocal laser scanning module TCS 4D (Leica), or a Zeiss LSM510 META (Zeiss). For detection of GFP, specimens were excited using an Argon 488 nm laser, and the BP 505–550 filter was used for detection. For mRFP1 and PI fluorescence, a helium neon laser (543 nm) was used for excitation in combination with a LP 560 filter. For capture and processing of confocal images the Zeiss LSM 510 META software and the Zeiss LSM Image Browser version 3.5.0.359 was used.

### Tandem Affinity Purification and Liquid Chromatography–Mass Spectrometry/Mass Spectrometry Analysis

Plant materials (30 g) of either cell suspensions harvested 7 days after subculturing (suspension experiments #1, #2, and #3 representing three biological replicates) or 12-day old seedlings (seedlings experiment #1) overexpressing TaMAB2-TAP recombinant protein (line EM4), were homogenized in liquid nitrogen. The same procedure was applied for SerRS-TAP (Kekez et al., 2016) used as a control sample. Crude protein extracts were prepared in an equal volume (w/v) of extraction buffer (50 mM Tris-HCl, pH 7.5, 150 mM NaCl, 10% (v/v) glycerol, 0.1% (v/v) Nonidet P-40, 1 mM DTT, 1 mM PMSF, 1x Roche complete protein inhibitor), at 4°C. The soluble protein fraction was obtained by centrifugation at 4°C. The extract was passed through a 0.45-µm filter. Purifications were performed as described by Rigaut et al. (1999), with some modifications. Briefly, total protein extract was incubated for 3 h at 4°C under gentle rotation with 300 µl of IgG-Sepharose 6 Fast Flow beads (GE Healthcare) pre-equilibrated with 10 ml of extraction buffer. IgG-Sepharose beads were transferred to a 1-ml Mobicol column (MoBiTec) and washed with 30 ml of immunoglobulin G (IgG) wash buffer (50 mM Tris-HCl, pH 8.0, 150 mM NaCl, 10% (v/v) glycerol, 0.1% Nonidet P-40, 1 mM DTT), and 10 ml of tobacco (*N. tabacum* L.) etch virus (TEV) buffer (50 mM Tris-HCl, pH 8.0, 150 mM NaCl, 0.1% (v/v) Nonidet P-40, 0.25 mM EDTA, 1 mM DTT). Bound complexes were eluted *via* AcTEV digest (50 units; Invitrogen) overnight at 4°C followed by wash with 500 µl of TEV buffer, and then 3 ml calmodulin binding buffer (50 mM Tris-HCl, pH 8.0, 150 mM NaCl, 0.1% (v/v) Nonidet P-40, 10 mM β-mercaptoethanol, 1 mM imidazole, 2 mM CaCl_2_, 1 mM magnesium acetate). The CaCl_2_ concentration of the IgG-eluted fraction was adjusted to 2 mM, and the fraction incubated for 1 h at 4°C under gentle rotation with 200 µl of calmodulin-agarose beads (Stratagene) pre-equilibrated with 10 ml of calmodulin binding buffer. Calmodulin-agarose beads were washed with 30 ml of calmodulin binding buffer and packed into a Mobicol column. Bound complexes were eluted with 2.5 ml of calmodulin elution buffer (50 mM Tris-HCl, pH 8.0, 150 mM NaCl, 0.1% (v/v) Nonidet P-40, 10 mM β-mercaptoethanol, 1 mM imidazole, 22 mM EGTA) and precipitated with trichloroacetic acid (20%, v/v). The protein pellet was washed with ice-cold acetone, redissolved in sample buffer, and separated on 4–12% gradient gel (Roth). Proteins were visualized with colloidal Coomassie Brilliant Blue staining. Parts of gels with proteins were cut into small (1 mm^2^) slices and further processed for MS analysis. Gel particles were washed three times with 5 mM ammonium bicarbonate (ABC) and 50% acetonitrile, dehydrated with 100% acetonitrile, reduced with 10 mM DTT in 20 mM ABC at 56°C, alkylated with 55 mM iodoacetamide and 20 mM ABC in the dark. After a two-step washing procedure with 5 mM ABC and 50% acetonitrile gel, slices were dehydrated in 100% acetonitrile and rehydrated in 50 µl of digest buffer containing 625 ng of trypsin (MS Gold; Promega). After 10 min 150 µl 20 mM ABC was added and proteins were digested at 37°C overnight. The resulting peptides were extracted with three serial washing steps: the first in 30% acetonitrile, 3% trifluoroacetic acid, the second in 80% acetonitrile and 0.5% acetic acid, and the third in 100% acetonitrile. Acetonitrile was evaporated using a vacuum centrifuge. Peptides were concentrated and purified with StageTips as described by Rappsilber et al. (2007). Briefly, StageTips were wetted by methanol and equilibrated by solution A (0.5% CH_3_COOH). Peptides were acidified by solution A, and loaded onto equilibrated tips. Salts were washed by solution A, and peptides were eluted from tips by solution B. Anhydrous acetonitrile was evaporated from desalted and purified peptides by vacuum centrifugation. Peptides were separated and then measured by a nanoscale HPLC system Easy-nLC (Proxeon Thermo) coupled to an LTQ-Orbitrap Discovery mass spectrometer (Thermo Scientific) through a nano-electrospray ionization source (Proxeon Thermo). Briefly, peptides were loaded onto C18 nanocolumn, made in-house by slurry packing PicoFrit capillaries (New Objective, 75 µm fused silica diameter, 10 µm tip diameter) with Luna 3 µm C18(2) material (Phenomenex). Peptides were eluted by a linear gradient of solution B in solution A (from 3 to 35% B in 45 min), and electrosprayed directly into the mass spectrometer. The mass spectrometer measured peptides in an orbitrap analyzer (one million ions at 30,000 resolution setting). In parallel, top 20 peptides were fragmented in a linear ion trap (3,000 ions) using dynamic exclusion to prevent recurring fragmentation of prominent peptides.

Raw data was processed by MaxQuant software as described by Cox and Mann (2008). UniProt *Arabidopsis* complete proteome set was searched using Andromeda (Cox et al., 2011) or X!Tandem (Craig and Beavis, 2004). UniProt ID numbers were obtained from mass spectrometry-based protein identification and searched against databases of the National Center for Biotechnology Information (NCBI, http://www.ncbi.nlm.nih.gov/protein) and the *Arabidopsis* Information Resource (TAIR, http://www.arabidopsis.org/) to annotate each gene. Target proteins were selected according to three criteria: firstly, they were presented by at least two peptides of which at least one was unique. Secondly, they were not presented in control experiment in which seryl-transfer ribonucleic acid (seryl-tRNA) synthetase was used as bait (proteins were considered as seryl-tRNA synthetase partners if they were presented by at least two peptides of which at least one was unique in any of the SerRS experiments). Thirdly, they were found in at least two independent experiments.

## Results

### TaMAB2 Belongs to the E3 Subclade of MATH-BTB Proteins

Wheat Ta*MAB2* was previously reported as a fertilization-induced gene, expressed exclusively in zygotes and two-celled proembryos (Leljak-Levanić et al., 2013). It displays significant similarity to core group MATH-BTB proteins from *Arabidopsis* (Weber and Hellmann, 2009) and ZmMAB1 from maize (Juranić et al., 2012), which was classified as an E2 group MATH-BTB protein (Juranić and Dresselhaus, 2014). To gain a deeper understanding about the phylogenetic relationship between TaMAB2 and other identified MATH-BTB proteins, we have searched the Ensembl Plants database against the *Triticum aestivum* proteome using BLASTp using TaMAB2 amino acid sequence as query. The search revealed 46 proteins in total, all of which were named according to the existing nomenclature as TaMAB1-46 (**Supplemental Table S2**). Amino acid sequences of 46 MATH-BTB proteins from wheat, 31 ones previously identified in maize (Juranić and Dresselhaus, 2014), 69 from rice (Gingerich et al., 2007), and 6 from *Arabidopsis* (Weber et al., 2005; Weber and Hellmann, 2009) were used to infer a maximum-likelihood phylogeny (**Supplemental Figures S1** and S2). It was previously reported that plant *MATH-BTB* genes cluster into a core clade and an expanded clade, which additionally clusters into five subclades named as E1–E5 (Gingerich et al., 2007; Juranić and Dresselhaus, 2014). Most MATH-BTB proteins of grasses clustered into the grass-specific expanded clade, with the exception of four genes from rice, four genes from wheat, and six genes of maize, which clustered into the core clade together with all six *Arabidopsis* genes. Proteins of the core clade are characterized by having multiple exons in the coding region, while proteins of the expanded clade most often contain a single exon within the coding region (Gingerich et al., 2007). Here, the four wheat MATH-BTB proteins which clustered into the core clade contain 4 or 5 exons (**Supplemental Table S2**). TaMAB2, on the other hand, contains only one exon, which supports its clustering into the expanded clade. Specifically, TaMAB2 clustered into E3 subclade, along with 13 other wheat (TaMAB3-10, 12, 17, 21, 22, and 46) and three rice proteins (OsMBTB6-8). Its most closest homologs are TaMAB7 and TaMAB17. Notably, this subclade did not contain any maize proteins. Gametophyte-specific ZmMAB1 of maize (Juranić et al., 2012) clustered into subclade E2 along with ZmMAB2-6 and four rice proteins (OsMBTB 29, 30, 31, 32). This subclade did not contain any wheat proteins. Presuming that functional similarity is preceded by similarity in amino acid sequence, Juranić and Dresselhaus (2014) suggested that ortholog and paralog proteins belonging to the same subclade likely share similar functions and prompted functional characterization of individual proteins from each subclade. None of the proteins clustering into the E3 subclade, where TaMAB2 belongs, have been functionally characterized to date.

### Overexpression of *TaMAB2* in *Arabidopsis thaliana* Causes Severe Growth Defects and Misorganization of Microtubule Bundles

**Figure 1:**
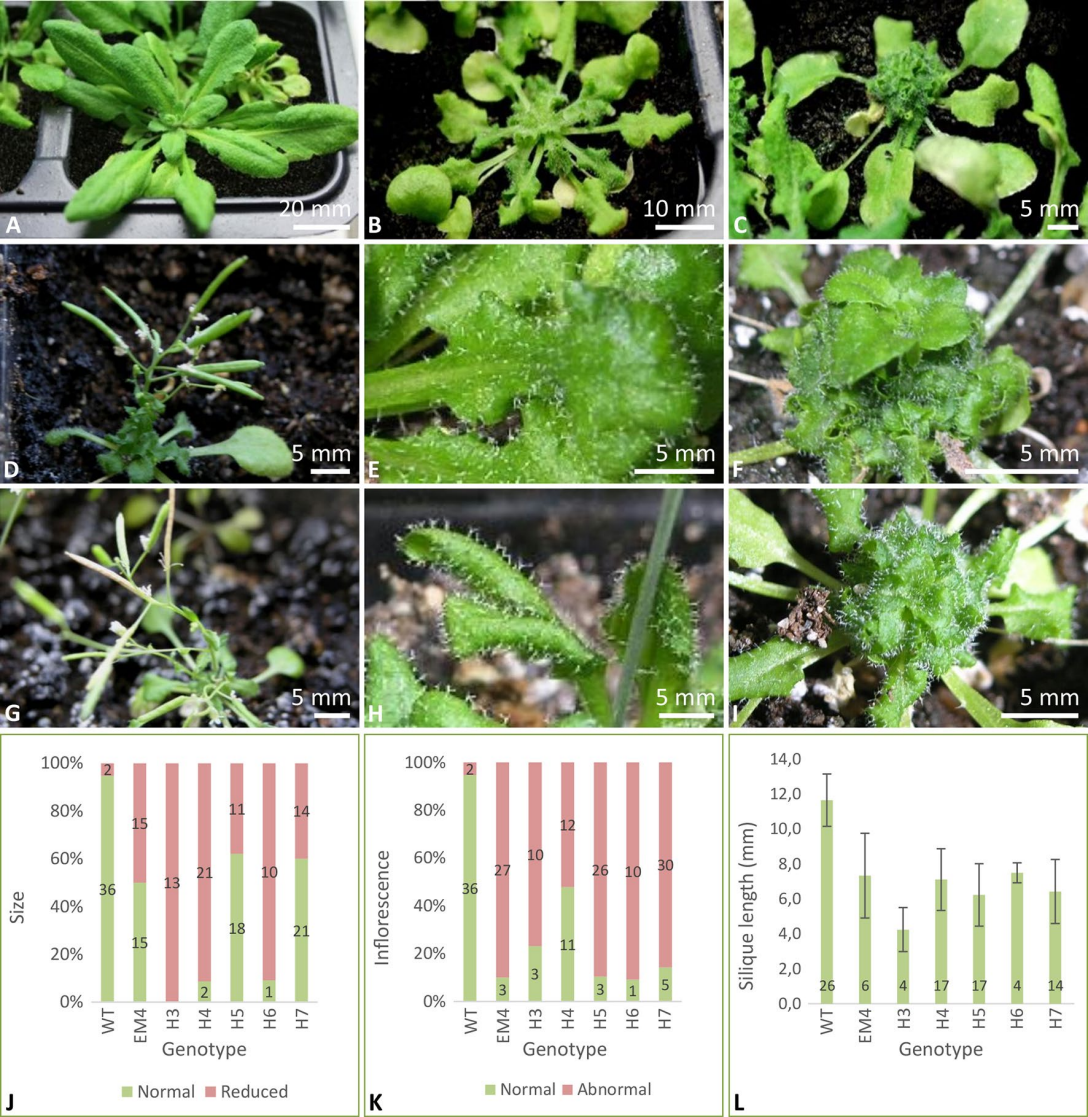
*Arabidopsis* plants overexpressing TaMAB2 show severe growth defects including small leaves, stalk elongation failures, inflorescences with few flowers, and short siliques. **(A)** Wild type plant, **(B**–**C)** TaMAB2-overexpression lines exhibiting weaker and stronger mutant phenotypes, respectively. Representative specimens of one homozygous line (EM4) are shown. **(D**, **G)** Two independent lines showing reduced inflorescence and silique elongation as well as **(E**, **H)** bundling of small and rugose leaves near the main stem **(F**, **I)** and a defect in leaf stalk elongation. Plants of two homozygous lines EM4 **(D**–**F)** and H4 **(G**–**I)** are shown. Overall growth parameters of wild type and six independent homozygous TaMAB2-overexpression lines are expressed as percentage of plants showing normal and reduced leaf rosette growth **(J)**, inflorescence elongation **(K)**, and silique length **(L)**. For each line, numbers in columns represent number of plants exhibiting respective phenotypes. Plants were photographed 37 days **(A**–**C**, **E**, **F**, **H**, and **I)** and 56 days **(D**, **G)** after sowing, respectively.

To obtain insights into the function of the first E3 subclade member TaMAB2, we initially applied an RNA interference (RNAi) approach to down-regulate *TaMAB2* in transgenic wheat. While we have successfully generated plants with downregulated fertilization-induced gene such as *TaEAL1* (Leljak-Levanić et al., 2013), we failed to generate *TaMAB2*-RNAi plants indicating that its function is essential and mutants are lethal. Therefore, we next switched to a heterologous system and overexpressed TaMAB2 in *A. thaliana* (either as *35S::TaMAB2-TAP* independent lines EM4, H3, H4, H5, H6, H7, *or 35S::TaMAB2-GFP* independent lines 80, 81, 82; **Supplemental Figure S3** *)* to investigate and assess TaMAB2 interference with growth and reproduction processes, and we aimed to isolate and identify possible substrates. While wild-type plants showed normal growth characteristics such as appearance of 14 rosette leaves followed by the formation of first flower buds and completion of flowering with fully developed siliques (Boyes et al., 2001), *TaMAB2*-overexpressing plants exhibited a number of growth defect phenotypes—regardless of the protein tag—which we classified into three types. While 16.6% of all *35S::TaMAB2-TAP* overexpressing plants were indistinguishable wild type plants (**Figure 1A**), 50% plants showed a weak mutant phenotype (**Figure 1B**) with only partial rosette growth: following the formation of the first 6–7 leaves, which appeared similar to wild type leaves, successive leaves of the rosette began to curl outwards, resulting in a rugose leaf blade (**Figures 1E, H**). Each new circle of leaves was smaller and more rugose than the previous one. Moreover, the leafstalks of these inner leaves failed to elongate, positioning younger leaves close to the main stalk (**Figure 1B**). Flower buds could not be observed at this stage. Thirty seven days after sowing, some further leaf growth could be observed, but leaves remained in a rosette formation. There was no stalk elongation and flowers did not emerge. Finally, 33.3% showed a strong phenotype (**Figure 1C**) exhibiting all the characteristics described above including pronounced leaf senescence. Thirty seven days after sowing, a dense bundle of leaves developed close to the plant stalk and subsequently failed to grow (**Figures 1F, I**). This growth regression began after the 6^th^ or 7^th^ rosette leaf was formed, with the bundle becoming visibly smaller and the rosette leaves wilting (**Figure 1I**). Depending on the transgenic line, the percentage of plants with reduced growth varied between 38% (line H5) and 100% (line H3) (**Figure 1J**).

**Figure 2:**
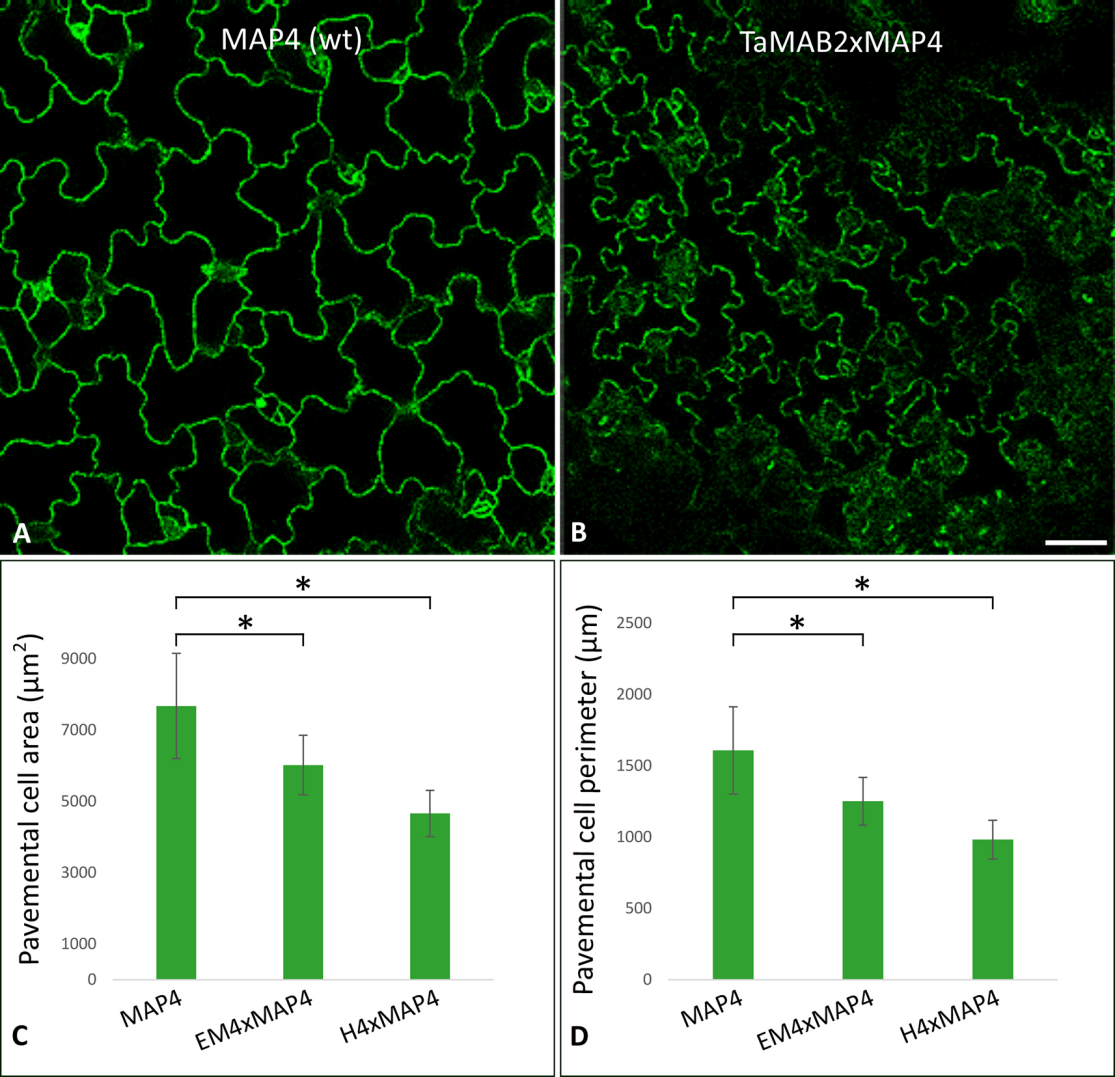
The size of epidermal pavement cells of *Arabidopsis* lines overexpressing *TaMAB2* is significantly reduced. A microtubule marker line (*MAP4*) was crossed with TaMAB2 overexpressing lines EM4 and H4 to generate microtubule marker lines overexpressing *TaMAB2* (*TaMAB2×MAP4*). **(A)** CLSM of epidermal pavement cells in cotyledons of 5 day-old seedlings in MAP4 that served as a wild type (wt) control and **(B)** TaMAB2xMAP4 crossbreed line, represented here by EM4xMAP4 crossbreed. **(C**, **D)** Cell area and perimeter is significantly reduced in *TaMAB2-*overexpressing crossbreed lines as indicated. For each line 50 cells of three individual seedlings were measured in three biological replicates and presented as mean values ± standard error (SE). Asterisk denotes significant difference at *P* < 0.05. Scale bar = 20 µm.

**Figure 3:**
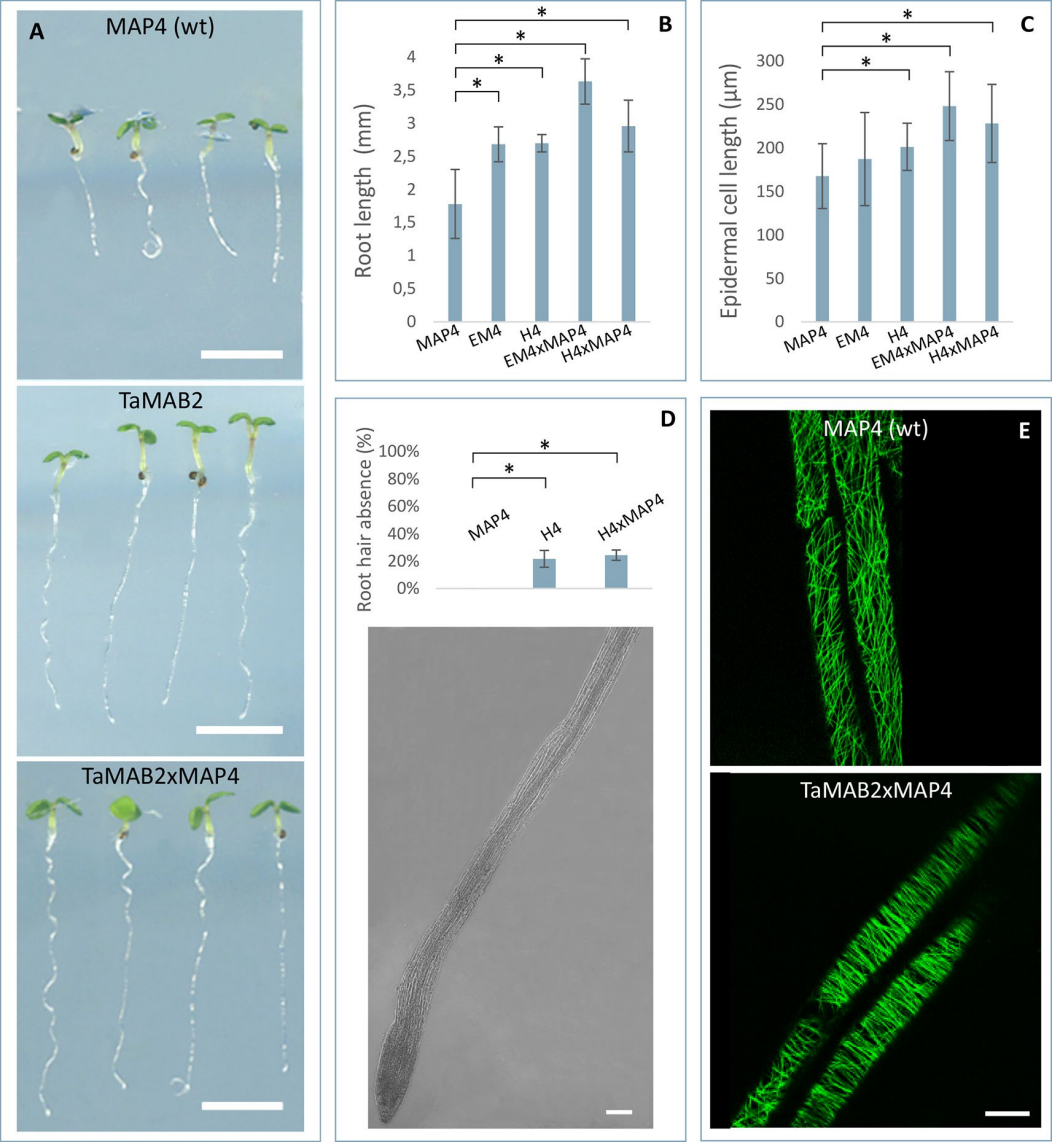
*TaMAB2*-overexpressing *Arabidopsis* lines display root defects including elongation of the primary root, partially absence of root hairs and disorganization of microtubule bundles. A microtubule marker line (*MAP4*) was crossed with TaMAB2 overexpressing lines EM4 and H4 to generate microtubule marker lines overexpressing *TaMAB2* (*TaMAB2×MAP4*). The MAP4 line was used as wild type (wt) control. **(A)** Primary root length in line MAP4, TaMAB2-overexpressing line (TaMAB2, represented by line EM4) and TaMAB2xMAP4 crossbreed (represented by EM4xMAP4). **(B)** Measurement of primary root length and **(C)** root epidermal cell length in the root hair appearance zone of 50 5-day-old seedlings in the lines indicated. Five cells per seedling were measured. Results are presented as mean values ± standard error (SE) of three biological replicates. Asterisk denotes significant difference at *P* < 0.05. **(D)** About 21–24% roots of *TaMAB2*–overexpressing line H4 lack root hairs. **(E)** Confocal laser scanning microscopy micrographs of microtubules in epidermal root cells in the region of first root hair appearance in a *MAP4* line and *TaMAB2xMAP4* line, respectively. EM4xMAP4 crossbreed is shown. Scale bars in **(A)** = 5 mm, and in **(D**, **E)** = 20 µm.

At long day conditions, *TaMAB2*-overexpressing *A. thaliana* plants successfully developed flower stalks and produced siliques. However, elongation of both stalks and siliques was reduced compared to wild type plants. Overexpressing mutant plants showed a stalk length reduced to about 1/10 of wild type stalk length (**Figures 1D, G**) in 52 to 91% plants of various lines (**Figure 1K**). Siliques were reduced from an average length of 12 mm in wild type plants to an average of 7 mm in overexpressing lines (**Figure 1L**). To elucidate the cause of the various growth defects, we next measured the size of selected cells in *TaMAB2*-overexpressing lines H4 and EM4 crossed with the MT marker line MAP4, further designated as EM4xMAP4 and H4xMAP4. The MT marker line used was chosen as it was shown previously that ectopic expression of GFB-MBD does not affect plant growth and the rate of root growth was reported to be similar between wild type and marker line (Granger and Cyr, 2001). Epidermal cell dimension analysis revealed significantly reduced expansion of epidermal pavement cells of cotyledons in both crossbreed lines (**Figures 2A, B**, represented by EM4 crossbreed). Compared to control (MT marker line MAP4), the average cell area of pavement cells was significantly reduced in both hybrid lines, 22% in EM4×MAP4 or 40% in H4×MAP4, which corresponded with significantly reduced cell perimeter of pavement cells (23% in EM4×MAP4 and 39% H4×MAP4 hybrid line) (**Figures 2C, D**). As opposed to reduced growth observed in shoot tissues, overexpression of *TaMAB2* was positively correlated with significantly induced elongation of primary roots. Compared to MAP4 control, the average root length of 5-day old hybrid transgenic seedlings generated from EM4 and H4 lines was significantly increased by 94 or 66%, respectively (**Figures 3A, B**). This is likely caused by an enlarged cell size, as the average length of epidermal root cells of hybrid transgenic plants was 51% (in EM4×MAP4) or 36% (in H4×MAP4) larger than in control (**Figure 3C**). Notably, 21–24% *TaMAB2*-overexpressing plants of H4 and H4×MAP4 lines did not develop trichoblasts in the epidermal layer of the primary root (**Figure 3D**). However, this was not shown for either EM4 or EM4×MAP4 line.

To investigate whether the growth defects and altered cell size of *TaMAB2*-overexpression plants are caused by defects in microtubule (MT) organization, we assessed the MT organization in the root apex and cells of the first root hair appearance zone in 5-day old seedlings of crossbreeds EM4×MAP4 and H4×MAP4. MT marker line was used as wild type control. Different types of MT arrays were detected in marker line including cortical MTs, preprophase band, mitotic spindle, and phragmoplast MTs. MTs of different lengths were oriented in various angles relative to the root axis from longitudinal to transversal (**Figure 3E**, upper) as previously described (Sugimoto et al., 2000; Panteris et al., 2015). By contrast, MTs of *TaMAB2-*overexpressing plants of both crossbreeds displayed extensive bundling and oblique positioning relative to the root axis (**Figure 3E**, lower, represented by EM4) indicating a role of TaMAB2 in mediating microtubule bundling and orientation during cell elongation.

### TaMAB2 Occurs in Foci and Co-Localizes With Microtubules

**Figure 4:**
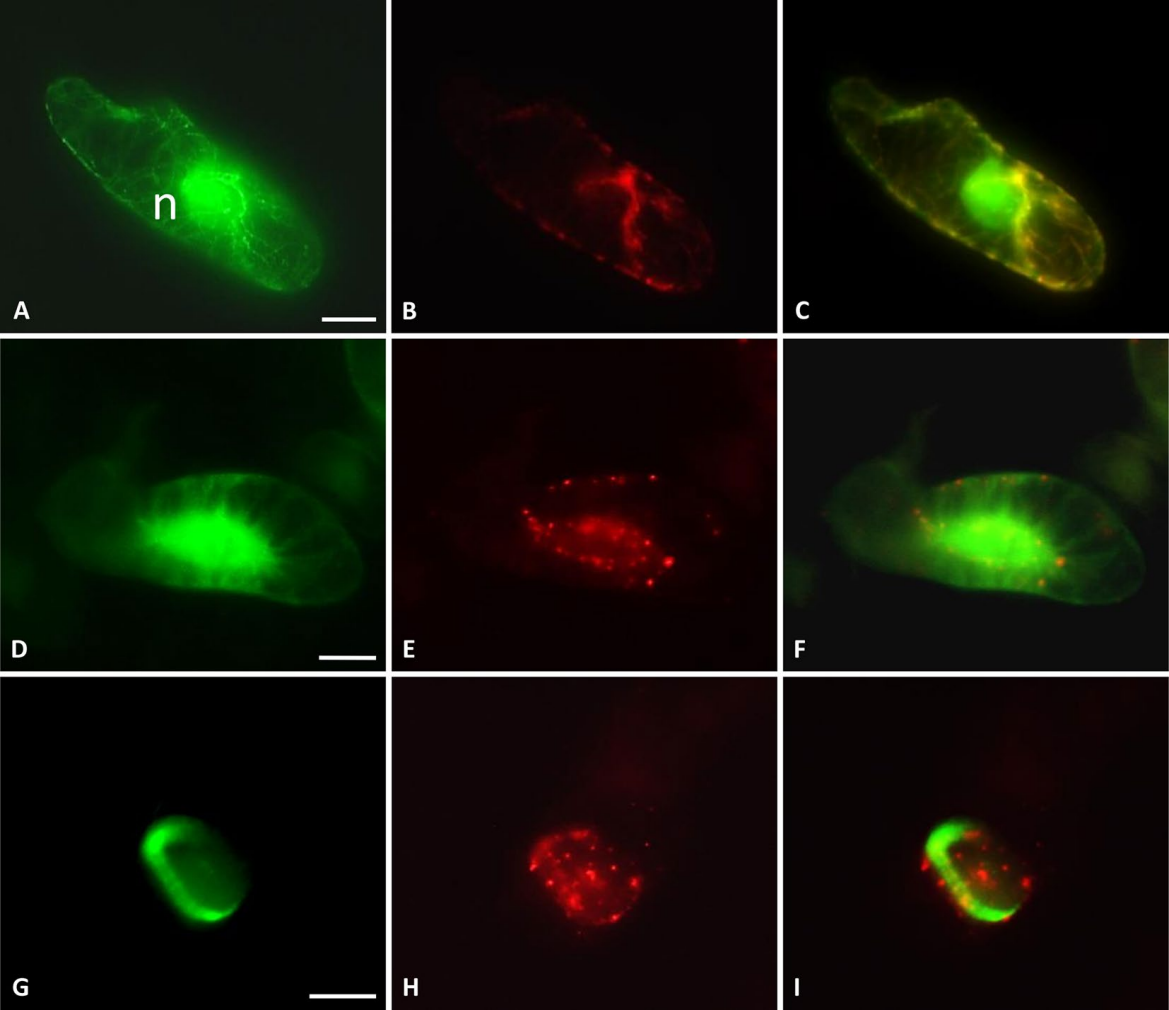
TaMAB2 accumulates in cytoplasmic foci during mitosis. Subcellular localization of TaMAB2 and microtubules (MTs) during the cell cycle after transient transformation of tobacco BY-2 cells with *35S::RFP-TaMAB2* (red) and a MT-labeling construct *35S::2xEGFP-MBD* (green). **(A**–**C)** Interphase, n - nucleus **(D**–**F)** G2-phase showing initiation of preprophase band formation, and **(G**–**I)** phragmoplast in telophase. **(A**, **D**, and **G)** are fluorescent images showing MTs, **(B**, **E**, and **H)** indicate TaMAB2, and **(C**, **F**, and **I)** are corresponding merged images. Experiment was repeated three times. Each time 100 labeled cells were analyzed. Scale bar = 20 µm.

Subcellular localization of TaMAB2 was previously investigated by transiently expressing a GFP-fused version of TaMAB2 (*Ubip::TaMAB2-GFP*) in tobacco BY-2 cells. In the majority of TaMAB2-GFP expressing cells, strong GFP signals accumulated as aggregates unilaterally around the nucleus with additional tiny green fluorescent spots present in the nucleus (Leljak-Levanić et al., 2013). Since EGFP has a tendency to self-aggregate (Krasowska et al., 2010) and many aggregates were unusually large, we cannot exclude that these represented artifacts. We therefore constructed an expression cassette of RFP-fused TaMAB2 (*35Sp::RFP-TaMAB2*). BY-2 cells were then co-transformed with this construct and the *35Sp::2xGFP-MmMBD* (GFP labeled microtubule BD of microtubule associated protein MAP4 from mouse) and were observed the subcellular localization of both proteins during the entire cell cycle. Cell cycle stages were distinguished according to their MT array organization as described in Rasmussen et al. (2013). Similar to previously reported TaMAB2-GFP localization (Leljak-Levanić et al., 2013), TaMAB2-RFP accumulated unilaterally around the nucleus and colocalized with MTs in nuclear vicinity in early interphase, with additional spreading along cortical MTs (**Figures 4A–C**). During late G2 phase, TaMAB2-RFP occurs in foci either around the nucleus or at the cell periphery always co-localizaing with MTs (**Figures 4D–F**). At the preprophase stage, TaMAB2-RFP occurs in foci surrounded by the preprohase band (**Figures 4G–I**).

**Figure 5:**
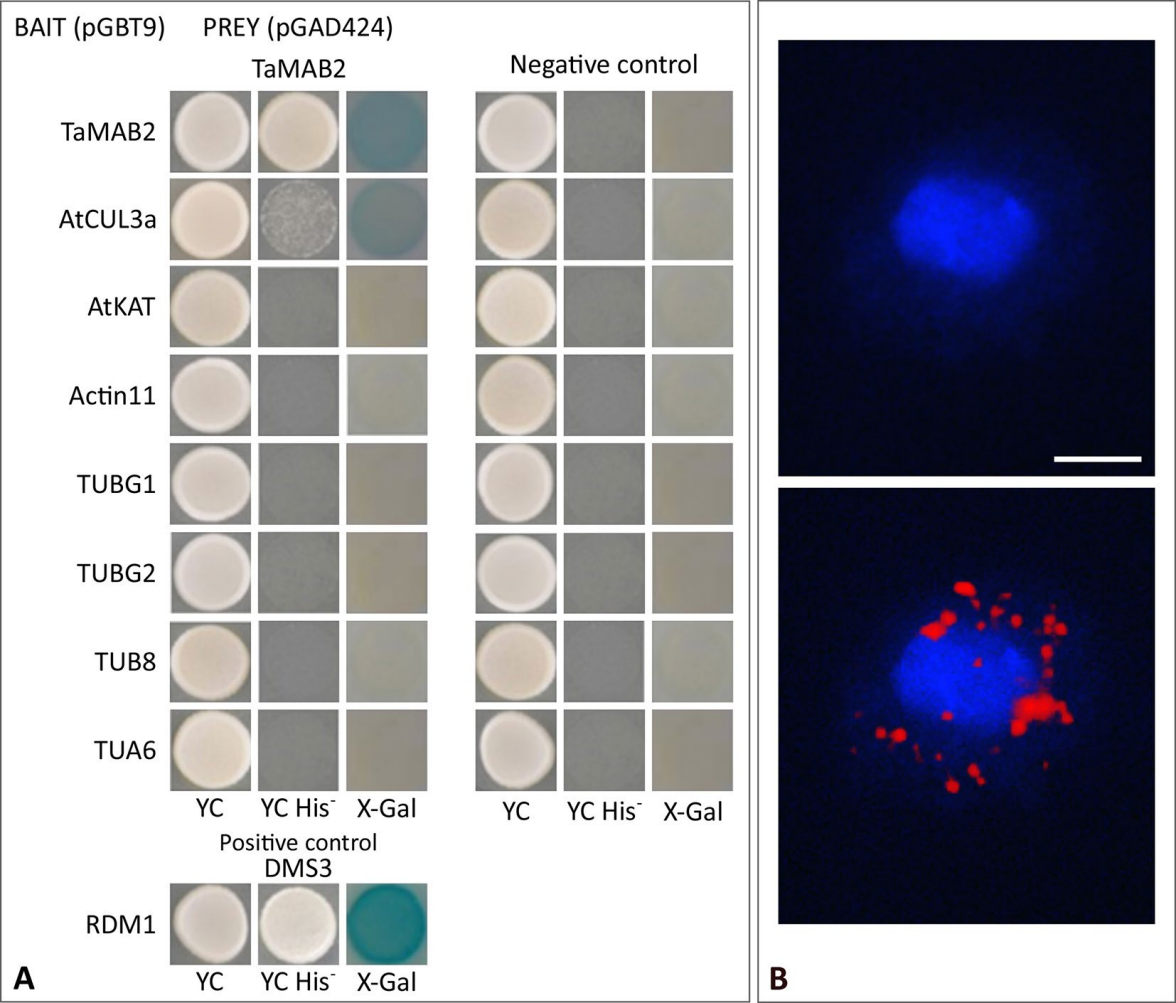
TaMAB2 likely accumulates in the cytoplasm in active dimeric E3 ligase complexes. This is indicated by dimerization as well as interaction with CUL3 and presence of ubiquitin. **(A)** Y2H protein interaction assay of TaMAB2 with AtCUL3 and cytoskeletal proteins AtKAT, AtACT11, AtTUBG1, AtTUBG2, AtTUA6, and AtTUB8 in His prototrophy and β-galactosidase assays. For each experiment six individual colonies were analyzed. Specificity of the bait construct was confirmed by co-transformation with empty prey vectors (negative control). DMS3-RDM1 interaction served as a positive control. **(B)** TaMAB2-green fluorescent protein (GFP) and ubiquitin co-localize to cytoplasmic complexes in transgenic *Arabidopsis* protoplasts as shown by Duolink *In Situ* proximity ligation assay (PLA). Primary antibodies against ubiquitin and GFP were combined with secondary antibodies emitting a red fluorescent signal [Texas (TX) red] when both antibodies are in close proximity. Protoplast nuclei were stained with 4′,6-diamidino-2-phenylindole and visualized under UV light (**B**, top) and merged with TX red signals (**B**, bottom). A minimum of 30 protoplasts emitting a PLA signal was analyzed in three independent experiments. Protoplasts of transgenic plants overexpressing TaMAB2-GFP (line 82) are shown. Scale bar = 20 µm.

To investigate whether TaMAB2 is capable to directly interact with tubulin and tubulin-associated proteins, we performed a yeast two-hybrid (Y2H) assay using full-length protein sequences of TaMAB2 as a prey as well as various tubulins as bait including TUBG1 (At3G61650), TUBG2 (At5G05620), TUB8 (At5G23860), and TUA6 (At4G14960) from *A. thaliana*. Additionally, we tested the tubulin severing factor katanin (KAT; At1G80350), which has been shown to interact with MEL26, a MATH-BTB protein in *C. elegans* (Pintard et al., 2003; Xu et al., 2003) and ZmMAB1 of maize (Juranić et al., 2012). None of the proteins selected showed a direct interaction with TaMAB2 (**Figure 5A**). However, this does not exclude the possibility that an interaction occurs indirectly mediated, for example, by other proteins or *via* post-translational modifications.

### TaMAB2 Appears to Accumulate in Active E3-Ligase Complexes in *Arabidopsis*

It was previously shown that members of the *Arabidopsis* and rice MATH-BTB protein family as well as ZmMAB1 interact with CUL3 proteins (Gingerich et al., 2005; Gingerich et al., 2007; Juranić et al., 2012) and likely act as substrate-specific adaptors in CUL3-based E3 ligases. Thus, we tested whether TaMAB2 is capable to form homodimers and to interact with full length CUL3a protein (AT1G26830). As shown in **Figure 5A**, TaMAB3 from wheat was not only able to form strong homodimers in a Y2H assay, it was also able to interact with CUL3a, although this interaction was weaker compared with homodimerization.

To study whether TaMAB2 also interacts with ubiquitin and whether ubiquitin is present in cytoplasmic foci containing TaMAB2, we perfomed a colocalization analysis in *A. thaliana* seedlings using Duolink *In Situ* PLA (OLINK Bioscience, Uppsala, Sweden). This assay detects both direct and indirect interactions *in situ* by using a pair of secondary antibodies labeled with complementary oligonucleotide probes (PLA probes) (Söderberg et al., 2006). When the two probes are in close proximity, they can hybridize and an individual fluorescent signal is formed through amplification (OLINK Bioscience). Here, anti-GFP and anti-ubiquitin primary antibodies were used. In transgenic protoplasts of 2-week-old seedlings, we found that TaMAB2-GFP colocalized with ubiquitin in foci around the nucleus (**Figure 5B**). Altogether, these interaction and colocalization studies suggest that TaMAB2 accumulates in cytoplasmic foci representing active E3-ligase complexes.

### Identification of Various eIF3 and eIF4 Subunits as TaMAB2 Interactors

The above described findings indicated that TaMAB2 targets substrates in *Arabidopsis* for ubiquitination. Finally, to identify such substrates, we established a tandem affinity purification protocol for *Arabidopsis* suspension cultures and seedlings. Mass spectrometry (MS) was performed to reveal the identity of direct and indirect TaMAB2 interaction partners. Proteins were extracted from 12-day old *Arabidopsis* seedlings (later designated as seedling experiment) and *Arabidopsis* cell suspension harvested 7 days after sub-culturing (suspension experiment #1, #2, and #3), respectively, overexpressing TaMAB2-TAP. Lines overexpressing an aminoacyl-tRNA synthetase (SerRS), a protein involved in protein synthesis (Perona and Gruic-Sovulj, 2014), served as a control to eliminate false positives.

Among all proteins determined in four experiments, only 10 proteins including TaMAB2, histones H2A and H4, as well as tubulin beta-2/beta-3 chains and ATP synthases alpha and beta were common for both tissue types. A subunit of the translation initiation factor 4 (eIF4A1) and actin 11 were identified only in cell suspension experiments, while three subunits of the translation initiation factor 3 (eIF3A, C, G) as well as chloroplast translation initiation factor 2 (IF2) and 30S ribosomal protein S9 appeared as putative TaMAB2 interactors in seedling (**Table 1**; see **Supplemental Tables S3** and **S4** for details). In summary, subunits of translation initiation factor complexes were absent in the SerRS control and strongly overrepresented in the TaMAB2 fractions suggesting that they are true substrates. Components of the *Arabidopsis* E3-ligase complex, AtCul3a, AtCul3b, and AtRBX1 shown to directly bind *Arabidopsis* BTB domain-containing proteins, including MATH-BTB proteins (Figueroa et al., 2005; Gingerich et al., 2005; Chen et al., 2013) were not detected in any of the experiments.

**Table 1:** List of TaMAB2 interactors identified after tandem affinity purification.

UniProt ID	Protein descriptions and functional categorization	Total peptides	PEP (log e)	Tissue
	**Cytoskeletal proteins**			
P29512	Tubulin beta-2/beta-3 chain	11	1.41E−06	Su/Se
P53496	Actin 11	10	(−20.4)	Su
	**Translation**			
P41376	Eukaryotic translation initiation factor 4A1	7	(−20.8)	Su
Q9LD55	Eukaryotic translation initiation factor 3 subunit A	6	4.53E−23	Se
Q9XJ27	30S ribosomal protein S9	4	1.99E−15	Se
O49160	Eukaryotic translation initiation factor 3 subunit C	2	5.60E−09	Se
Q9SHI1	Translation initiation factor IF-2	3	1.10E−12	Se
F4J6A1	Eukaryotic translation initiation factor 3 subunit G	2	1.67E−05	Se
	**DNA/chromatin modification**			
P59259	Histone H4	12	1.75E−34	Su/Se
O23628	Histone H2A	4	2.03E−04	Su/Se
P59169	Histone H3	2	6.25E−04	Se
	**Photosynthesis/chloroplast related**			
P19366	ATP synthase subunit beta, chloroplastic	25	1.75E−29	Su/Se
P56757	ATP synthase subunit alpha, chloroplastic	14	4.30E−28	Su/Se

Protein extracts were generated from TaMAB2-TAP-overexpressing suspension (Su) cultures (three biological replicates) and seedlings (Se), respectively. See **Supplemental Tables S3 and S4** for more details. Proteins were considered as potential interaction partners of TaMAB2 if they were presented by at least two peptides of which at least one was unique in at least two independent experiments (either with cell suspension or seedlings). Moreover, proteins were excluded if they were present in control experiment in which seryl-tRNA synthetase was used as a bait. UniProt protein identifiers are shown at the left, total number of peptides, error probability (expressed as posterior error probability – PEP or log e values), and tissue type used at the right.

## Discussion

The MATH-BTB protein family is common in both animals and plants, but not in fungi (Juranić and Dresselhaus, 2014). Its members assemble with CUL3 and RBX1 into E3-ligases to promote selective ubiquitination of various substrate proteins. A distinctive feature of the MATH-BTB family is its extensive expansion in the grasses and nematodes (Stogios et al., 2005; Juranić et al., 2012). As proposed by Gingerich et al. (2007), the MATH-BTB families of rice and other grasses are likely rapidly changing and expanding to cope with targets that may also be rapidly changing due to grasses’ accelerated evolution (Salse et al., 2009). In this work, with the identification of 45 additional wheat MATH-BTB proteins related to previously reported TaMAB1-3, we detected a similar degree of MATH-BTB family expansion in wheat. Similar to other grasses, wheat MATH-BTB proteins clustered primarily into the grass-specific expanded clade with only four proteins clustering into the core clade. TaMAB2 analyzed here in more detail clustered into the E3 subclade. To date, functional studies or protein-protein interaction analyses have not been reported with any of the wheat MATH-BTB proteins likely due to technical difficulties to perform functional analyses with this species. Our RNAi approach to down-regulate *TaMAB2* also failed. This could have been caused due to a key role of TaMAB2 during early embryogenesis or due to simultaneous down-regulation of *TaMAB7* and *TaMAB17* which share high sequence similarity with TaMAB2 and may have redundant roles. Probably even more TaMABs of subclade E3 consisting of 14 members in wheat might be affected by an RNAi approach. An extensive clusters of regularly interspaced short palindromic repeats (CRISPR)/Cas9 approach could be a solution for future studies, but this will remain challenging and very time-consuming considering the gene redundancy discussed above.

An overexpression approach in an “easy” plant species such as *Arabidopsis* could contribute and support studies in “difficult” species such as wheat and has thus been applied in numerous studies. Also in this study, we found that overexpression of TaMAB2 caused severe growth defects indicating that the wheat protein interacted with *Arabidopsis* proteins and pathways. Especially remarkable was the finding that cells in overexpressing plants were significantly smaller, partly elongated and MTs misorganized. However, introduction of a novel MATH-BTB protein into a heterologous plant system implies a possibility that the overexpressed TaMAB2 in our transgenic lines competed with endogenous MATH-BTBs (or even other BTB proteins) for Cul3 binding, thus disrupting endogenous BTB/CUL3 E3 complexes. Several arguments could be made against this hypothesis. Firstly, the primary root length of TaMAB2 overexpression lines was increased compared to control, whereas the opposite is described for CUL3 hypomorphic double mutant (cul3^hyp^) lines, in which both Cul3a and Cul3b are nonfunctional (Thomann et al., 2009). Similarly, primary root length is reduced in lines with downregulation of all six AtBPMs (6xami*BPM*), as well as in lines with overexpression of MATH-domain of AtBPM1 (Chen et al., 2013). Overexpression of full-length AtBPM1 also induced a number of different phenotypes in mutant plants, such as early flowering, curving of rosette leaves in a counter clockwise direction, shorter petioles, and wider leaf blades (Škiljaica et al., unpublished), none of which were observed in TaMAB2 overexpressors. Together, these comparisons indicate that overexpression of TaMAB2 induced a highly specific phenotype and thus argue against the hypothesis that the mutant phenotype was caused merely by disruption of endogenous mechanisms. Finally, the absence of Cul3 in complex with TaMAB2 as part of the pull down assay suggests that the *Arabidopsis* Cul3 protein had much higher affinity for endogenous BTB proteins, and that the function of endogenous BTB/CUL3 E3 ligases remained largely intact in our overexpression lines. Nevertheless, because the *TaMAB2* gene is expressed only in the wheat zygote and 2-celled proembryo (Leljak-Levanić et al., 2013), the true physiological functions of the TaMAB2 protein can only be assessed in these developmental stages, and not globally. Thus, although the growth defects observed in our *Arabidopsis* model offer valuable insights into potential functions of TaMAB2, they cannot be extrapolated as genuine evidence of its physiological effects in wheat.

The only MATH-BTB protein functionally described in a grass species is ZmMAB1 belonging to the E2 subclade of MATH-BTB proteins (Juranić et al., 2012). Similar to its animal ortholog CeMEL26 (Pintard et al., 2003) and TaMAB2, ZmMAB1 is highly expressed in the maize zygote, and involved in MT-mediated processes such as correct chromosome segregation, spindle formation during meiosis and correct nuclei separation. Additionally, it interacted with the MT-severing factor katanin and the E3-ligase component CUL3. Although we did not find a direct interaction of TaMAB2 with katanin or various tubulins in a Y2H assay, we cannot rule out the possibility that it interacts indirectly. This hypothesis is supported by the finding that tubulin was detected as a target by tandem affinity purification and that TaMAB2 colocalized with MTs in interphase nuclei. Moreover, in the cell suspension pull down experiments, we detected actin11, an actin isoform expressed in female reproductive tissues and embryos of *Arabidopsis* (Huang et al., 1997), which overlaps with the site of expression of TaMAB2 in wheat. As indicated by its interaction with CUL3a, homodimerization and the presence of ubiquitin in cytoplasmic TaMAB2-containing foci, TaMAB2 is potentially another MATH-BTB protein discovered to act as the substrate-selective component of active E3 ligase complexes. Future experiments could reveal whether TaMAB2 interacts with cytoskeletal components in the zygote as its physiologically relevant site of function, and whether this interaction is carried out within an E3 ligase complex.

A very unexpected finding was the identification of various subunits of translation initiator complexes as putative TaMAB2 substrates. This includes the DEAD-box RNA helicase eIF4A1, which is known to unwind the secondary structure within the mRNA 5’ untranslated region (5’-UTR) and anchors the 43S pre-initiation ribosome complex allowing it to scan for the start codon. This interaction is also a rate limiting step ensuring differential translation (Bush et al., 2015). In the *Arabidopsis* genome, two genes encode eIF4A, *EIF4A1* and *EIF4A2*, but only *EIF4A1* knock-out mutants exhibit altered growth and cell size in a cell type-specific manner (Bush et al., 2015; Bush et al., 2016). These growth and cell size defects are partly similar to *TaMAB2*-overexpression effects supporting the conclusion that eIF4A, which is highly conserved in eukaryotes (Bush et al., 2015), is potentially a TaMAB2 substrate. Moreover, regulation of helicase activity of eIF4A is mediated by ubiquitination/deubiquitination (Li et al., 2018) further supporting our finding. eIF3 was identified as another interactor of TaMAB2. eIF3 is the largest eukaryotic initiation factor considered to be a ‘master regulator’ of initiation (Korostelev, 2014) acting downstream of eIF4 (Burks et al., 2001; Xia et al., 2010). It is involved in assembling the eIF2-GTP-Met-tRNAi*Met* ternary complex and recruiting it to the 40S subunit, recruiting mRNA to the 43S pre-initiation complex, and scanning for and recognizing AUG start codons (Burks et al., 2001; Kawaguchi and Bailey-Serres, 2002; Siridechadilok et al., 2005; Hinnebusch, 2006). The complex is comprised of 11 subunits, four of which were identified as potential interactors of TaMAB2 (eIF3A, C, and G). Subunits A, C, and G are part of the functional eIF3 core that is conserved in all eukaryotes (Asano et al., 1997) and necessary for translation initiation at all stages of development (Zhou et al., 2005). It is thus not surprising that degradation or any type of modulation of eIF3 complex activity by TaMAB2 overexpression leads to severe growth and developmental defects. Nevertheless, by using *Arabidopsis* as the host plant for overexpression of TaMAB2, we have introduced an expanded clade protein into a species with only core clade MATH-BTB members. This approach offered no assurance that TaMAB2 would successfully bind the *Arabidopsis* homologs of its true wheat interactors, or that they are even present in *Arabidopsis*. However, despite the restriction in drawing direct conclusions about true TaMAB2-interactors from this assay, our results offer important insights and direction for future research.

Neither Cul3A nor Cul3B were detected in complex with TaMAB2 in the tandem affinity assays, possibly due to Cul3 proteins forming a higher affinity bond with endogenous AtBPM proteins of the core group. It is also possible that TaMAB2 acts *via* a cullin3-independent mechanism. Participation in cullin-dependent and cullin-independent interactions has been previously shown for MEL-26, a MATH-BTB protein of *C. elegans* (Luke-Glaser et al., 2005). Future research on TaMAB2 should aim to reveal whether a particular TaMAB2 interactor is a target for ubiquitin-mediated degradation. Demonstrating a decrease in protein levels of putative TaMAB2 targets (such as eIF3 or eIF4A) in TaMAB2 overexpressors compared to wild type would be a good starting point in answering this question.

TaMAB2 clustered into the E3 subclade of the expanded clade. According to Salse et al. (2009), the expansion of MATH-BTB proteins observed in grasses is likely an adaptive measure to cope with their rapidly evolving targets. Therefore, the expected interactors of TaMAB2 would be rapidly evolving and grasses-specific proteins, as opposed to proteins involved in fundamental processes such as cytoskeletal regulation and translation. We propose that the specificity of targeting within the expanded clade is achieved not according to a “type” of a cell process, but its spatial and temporal regulation. The gene-expression profiles of grasses’ MATH-BTB genes described to date suggest a tightly regulated expression and turnover. For instance, ZmMAB1 of maize is expressed only in the male and female gametophyte and the zygote (Juranić et al., 2012) and expression of its closest paralogs ZmMAB2 and ZmMAB3 is germline-specific (Juranić and Dresselhaus, 2014). Meanwhile, TaMAB1 of wheat is expressed exclusively in the egg cell and TaMAB2 in the zygote and two-celled proembryo (Leljak-Levanić et al., 2013). Both ZmMAB1 and TaMAB2 are members of the expanded clade, yet both seem to be involved in fundamental processes, such as cytoskeletal regulation during the meiosis-to-mitosis transition in both the male and female gametophyte, as shown for ZmMAB1 (Juranić et al., 2012), or the possible interaction of TaMAB2 with components of the cytoskeleton and translation initiation complex (this work). Thus, the assumed specificity of expanded clade proteins of grasses possibly lies in the very short window of time when they are active. Interestingly, the expression patterns of ZmMAB1-3, which are all germline-specific E2 subclade proteins, slightly differ from one another and the genes appear to have a single locus origin (Juranić and Dresselhaus, 2014). The authors thus suggested a hypothesis that all E2 subclade members are expressed in germ lines and have a function in reproduction. Following a similar scenario, the E3 subclade proteins could be performing their own distinct roles, possibly in egg cells, zygotes, and at the onset of embryogenesis. A detailed gene expression pattern analysis of other members of the E3 subclade (and other subclades) could offer insights into the evolutionary purpose of MATH-BTB expansion in grasses, and reveal its possible connection to a spatial and temporal regulation of otherwise global mechanisms essential for plant growth and development.

Transgenic wheat lines have to be established now using CRISPR/Cas technology to knock-out *TaMAB2*, and likely also its sister genes *TaMAB7* and *TaMAB17* to elucidate is detailed function at the onset of wheat embryogenesis. Moreover, biochemical assays could help to investigate post-translational ubiquitination *via* TaMAB2-containing E3 ligases on the activity of translation initiation complexes. These are very challenging future experiments, but they could shed light on the functions of TaMAB2 and possibly other members of the MATH-BTB family in wheat during the very earliest stages of development.

## Data Availability Statement

The mass spectrometry proteomics data have been deposited to the ProteomeXchange Consortium (http://proteomecentral.proteomexchange.org) via the PRIDE partner repository (Vizcaino et al. 2013) with the dataset identifier PXD014358.

## Author Contributions

DL, TD, and SS designed the study. DL interpreted the results, carried out the localization and colocalization experiments, and analyzed MS spectrometry data. NB and GR carried out the MS spectrometry and AŠ as well as MK performed phenotypic analysis. AŠ carried out phylogenetic analysis and AŠ, MM, and MJ Y2H analysis. NM perfomed analysis of MT dynamics. DL wrote the paper with support of TD and input from all authors.

## Funding

This work was supported by grants from the Alexander von Humboldt-Foundation (to TD and DL), the German Research Council DFG *via* SFB960 (to TD and SS), by the Croatian Ministry of Science, Education and Sport to DL (Project No. 119-1191196-1225) by Croatian Science Foundation to DL (HRZZ, IP-2016-06-6229) and an institutional project financed by the University of Zagreb.

## Conflict of Interest

The authors declare that the research was conducted in the absence of any commercial or financial relationships that could be construed as a potential conflict of interest.

## Abbreviations

MATH, meprin and TRAF homology domain; BTB, Bric à brac/tramtrack/homology domain; TaMAB2, Triticum aestivum MATH-BTB protein 2; Cul3, Arabidopsis thaliana cullin3 protein; TUA6, Arabidopsis thaliana tubulin alpha chain 6; TUB8, Arabidopsis thaliana tubulin alpha chain 6; KAT, Arabidopsis thaliana katanin; SerRS, Arabidopsis thaliana seryl-tRNA synthetase; eIF, eukaryotic translation initiation factor; MT, microtubules; BY-2, Nicotiana tabacum cv. bright yellow—two cells.
