# Supplementary material for: The MATH-BTB Protein TaMAB2 Accumulates in Ubiquitin-Containing Foci and Interacts With the Translation Initiation Machinery in Arabidopsis
Source: Front Plant Sci. 2019 Nov 22;10:1469. doi: 10.3389/fpls.2019.01469 (PMC6883508; doi:10.3389/fpls.2019.01469)
Supplement: Supplementary file 1 [file Presentation_1.pptx]

## Slide 1
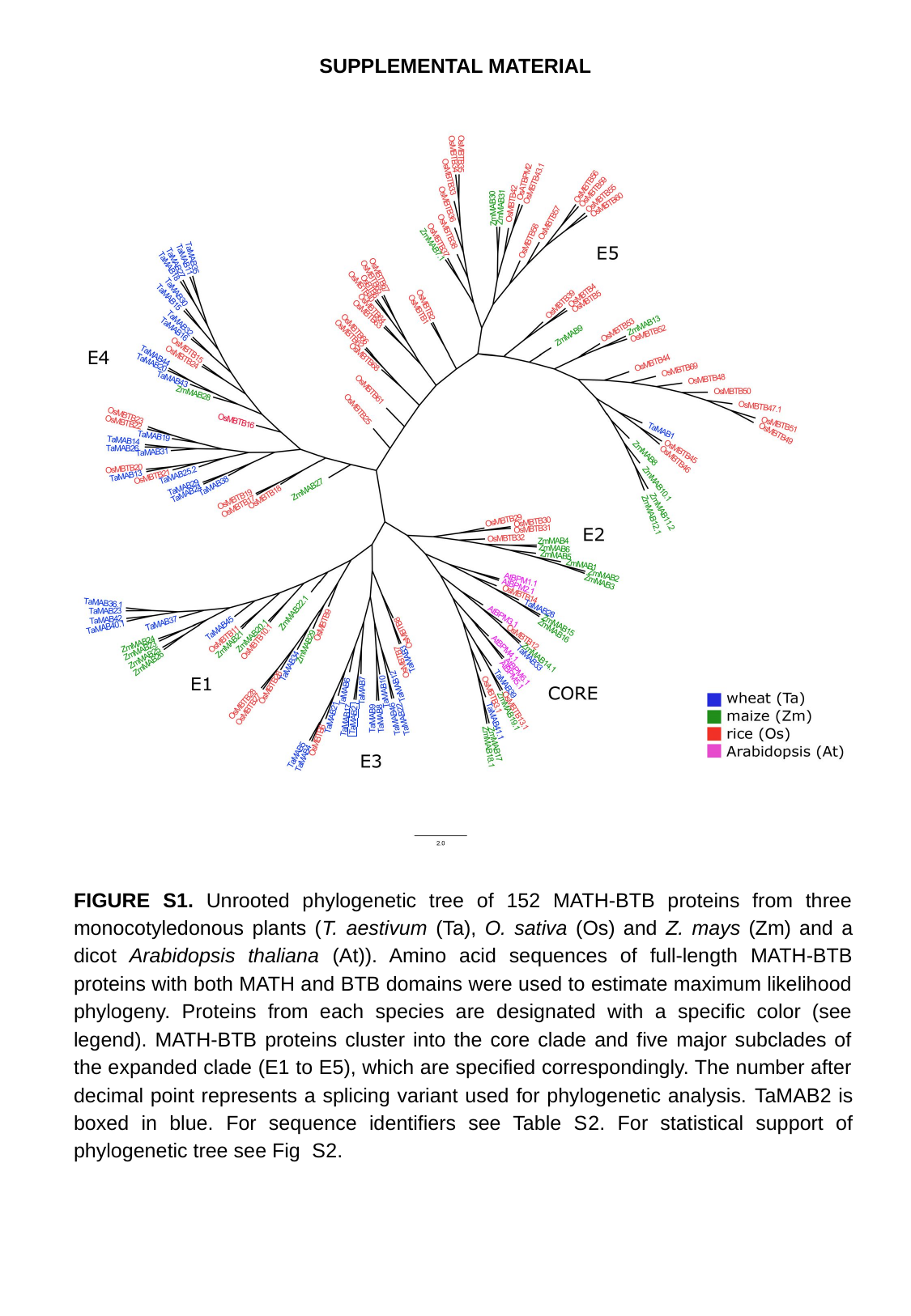

SUPPLEMENTAL MATERIAL
FIGURE S1. Unrooted phylogenetic tree of 152 MATH-BTB proteins from three monocotyledonous plants (T. aestivum (Ta), O. sativa (Os) and Z. mays (Zm) and a dicot Arabidopsis thaliana (At)). Amino acid sequences of full-length MATH-BTB proteins with both MATH and BTB domains were used to estimate maximum likelihood phylogeny. Proteins from each species are designated with a specific color (see legend). MATH-BTB proteins cluster into the core clade and five major subclades of the expanded clade (E1 to E5), which are specified correspondingly. The number after decimal point represents a splicing variant used for phylogenetic analysis. TaMAB2 is boxed in blue. For sequence identifiers see Table S2. For statistical support of phylogenetic tree see Fig S2.

## Slide 2
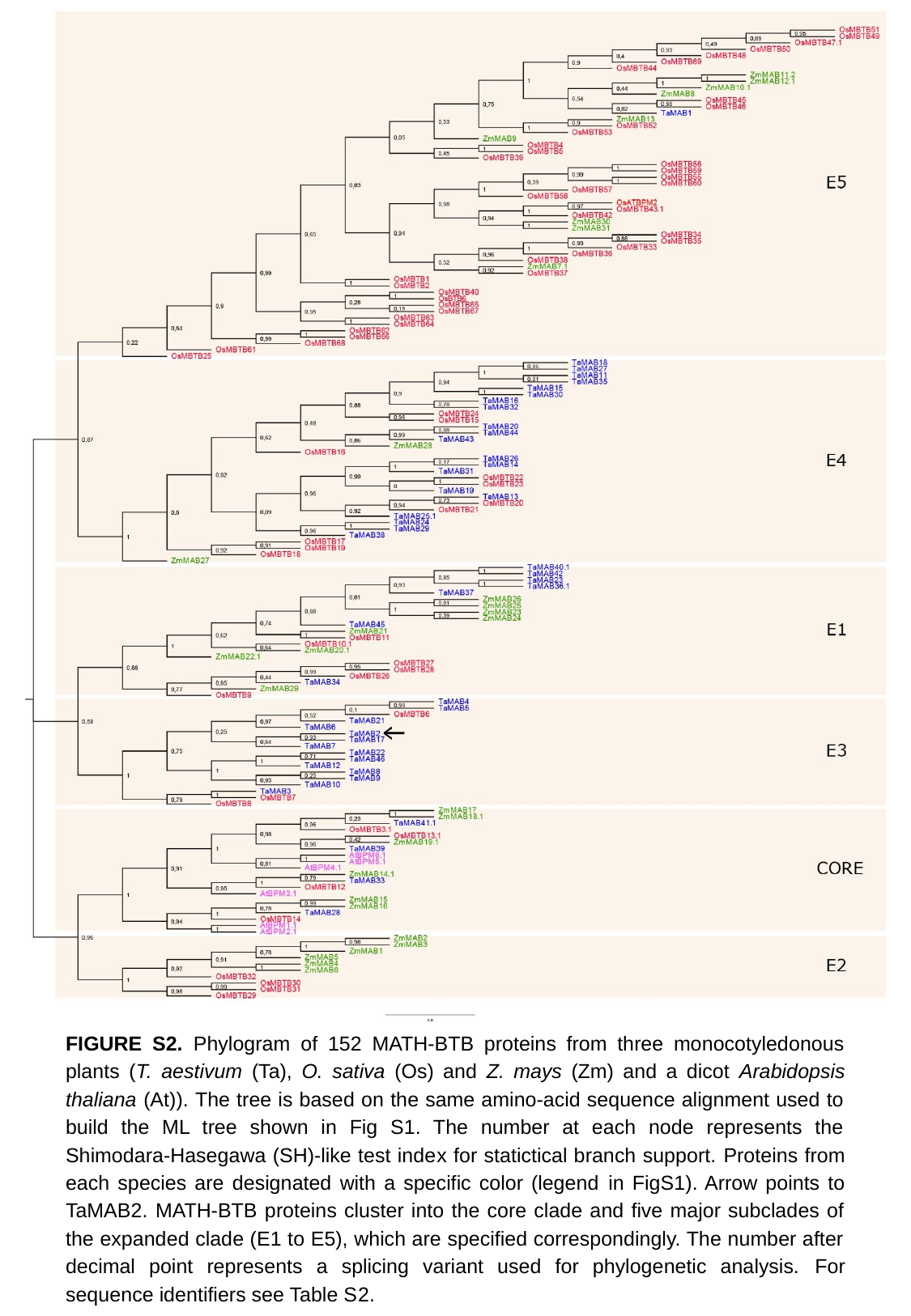

FIGURE S2. Phylogram of 152 MATH-BTB proteins from three monocotyledonous plants (T. aestivum (Ta), O. sativa (Os) and Z. mays (Zm) and a dicot Arabidopsis thaliana (At)). The tree is based on the same amino-acid sequence alignment used to build the ML tree shown in Fig S1. The number at each node represents the Shimodara-Hasegawa (SH)-like test index for statictical branch support. Proteins from each species are designated with a specific color (legend in FigS1). Arrow points to TaMAB2. MATH-BTB proteins cluster into the core clade and five major subclades of the expanded clade (E1 to E5), which are specified correspondingly. The number after decimal point represents a splicing variant used for phylogenetic analysis. For sequence identifiers see Table S2.

## Slide 3
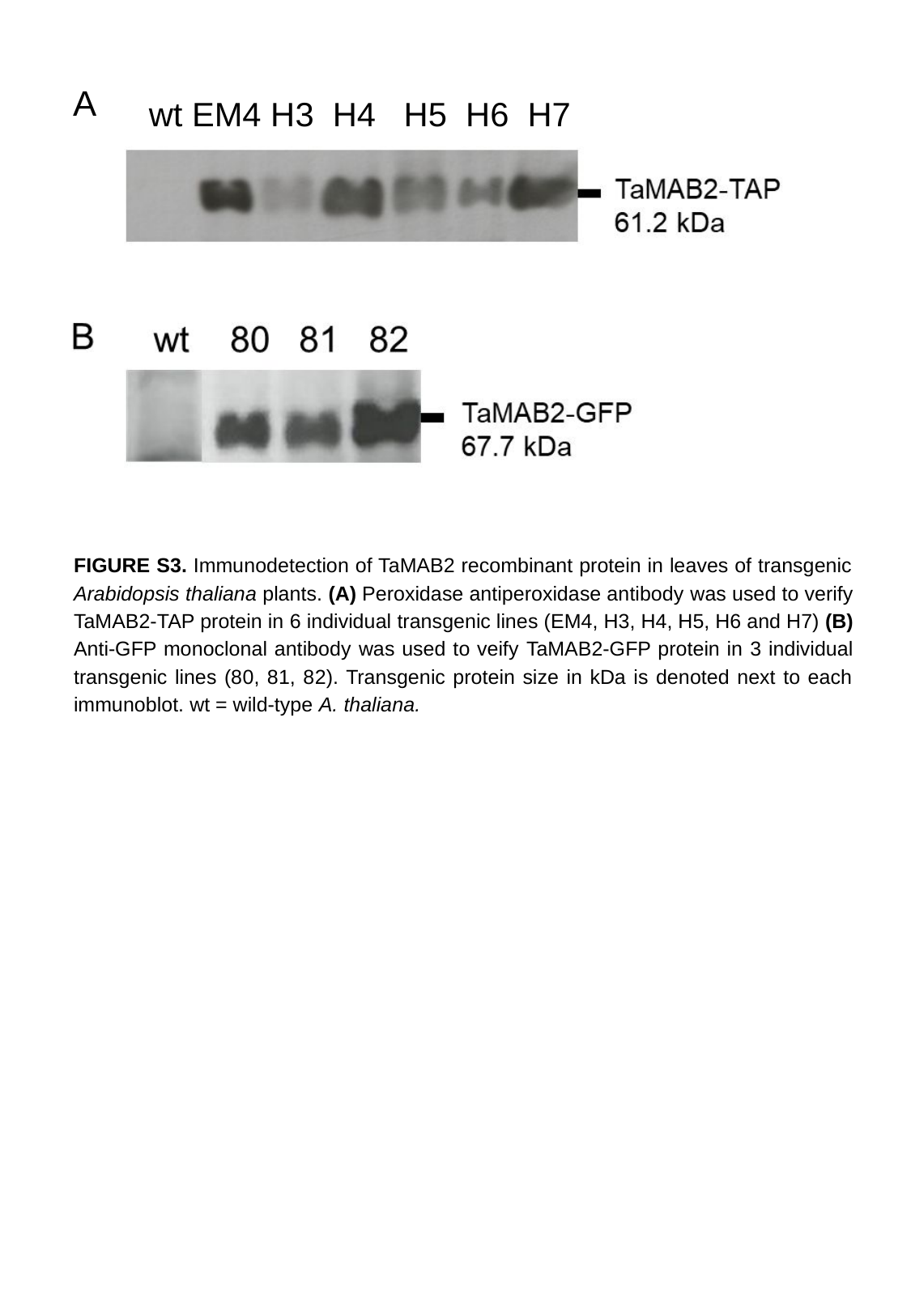

A
wt EM4 H3 H4 H5 H6 H7
FIGURE S3. Immunodetection of TaMAB2 recombinant protein in leaves of transgenic Arabidopsis thaliana plants. (A) Peroxidase antiperoxidase antibody was used to verify TaMAB2-TAP protein in 6 individual transgenic lines (EM4, H3, H4, H5, H6 and H7) (B) Anti-GFP monoclonal antibody was used to veify TaMAB2-GFP protein in 3 individual transgenic lines (80, 81, 82). Transgenic protein size in kDa is denoted next to each immunoblot. wt = wild-type A. thaliana.

## Slide 4
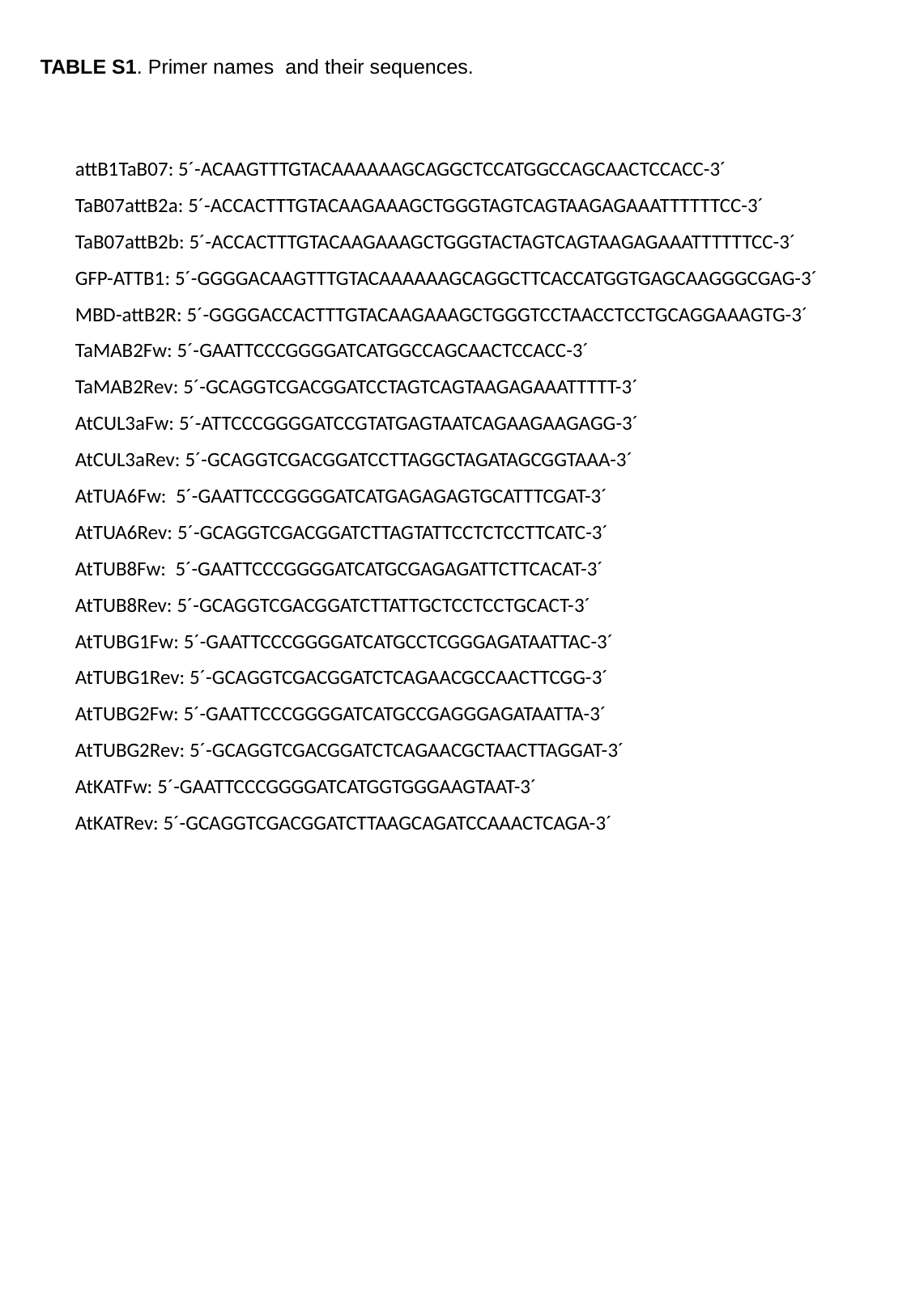

TABLE S1. Primer names and their sequences.
attB1TaB07: 5´-ACAAGTTTGTACAAAAAAGCAGGCTCCATGGCCAGCAACTCCACC-3´
TaB07attB2a: 5´-ACCACTTTGTACAAGAAAGCTGGGTAGTCAGTAAGAGAAATTTTTTCC-3´
TaB07attB2b: 5´-ACCACTTTGTACAAGAAAGCTGGGTACTAGTCAGTAAGAGAAATTTTTTCC-3´
GFP-ATTB1: 5´-GGGGACAAGTTTGTACAAAAAAGCAGGCTTCACCATGGTGAGCAAGGGCGAG-3´ MBD-attB2R: 5´-GGGGACCACTTTGTACAAGAAAGCTGGGTCCTAACCTCCTGCAGGAAAGTG-3´
TaMAB2Fw: 5´-GAATTCCCGGGGATCATGGCCAGCAACTCCACC-3´
TaMAB2Rev: 5´-GCAGGTCGACGGATCCTAGTCAGTAAGAGAAATTTTT-3´
AtCUL3aFw: 5´-ATTCCCGGGGATCCGTATGAGTAATCAGAAGAAGAGG-3´
AtCUL3aRev: 5´-GCAGGTCGACGGATCCTTAGGCTAGATAGCGGTAAA-3´
AtTUA6Fw: 5´-GAATTCCCGGGGATCATGAGAGAGTGCATTTCGAT-3´
AtTUA6Rev: 5´-GCAGGTCGACGGATCTTAGTATTCCTCTCCTTCATC-3´
AtTUB8Fw: 5´-GAATTCCCGGGGATCATGCGAGAGATTCTTCACAT-3´
AtTUB8Rev: 5´-GCAGGTCGACGGATCTTATTGCTCCTCCTGCACT-3´
AtTUBG1Fw: 5´-GAATTCCCGGGGATCATGCCTCGGGAGATAATTAC-3´
AtTUBG1Rev: 5´-GCAGGTCGACGGATCTCAGAACGCCAACTTCGG-3´
AtTUBG2Fw: 5´-GAATTCCCGGGGATCATGCCGAGGGAGATAATTA-3´
AtTUBG2Rev: 5´-GCAGGTCGACGGATCTCAGAACGCTAACTTAGGAT-3´
AtKATFw: 5´-GAATTCCCGGGGATCATGGTGGGAAGTAAT-3´
AtKATRev: 5´-GCAGGTCGACGGATCTTAAGCAGATCCAAACTCAGA-3´

## Slide 5
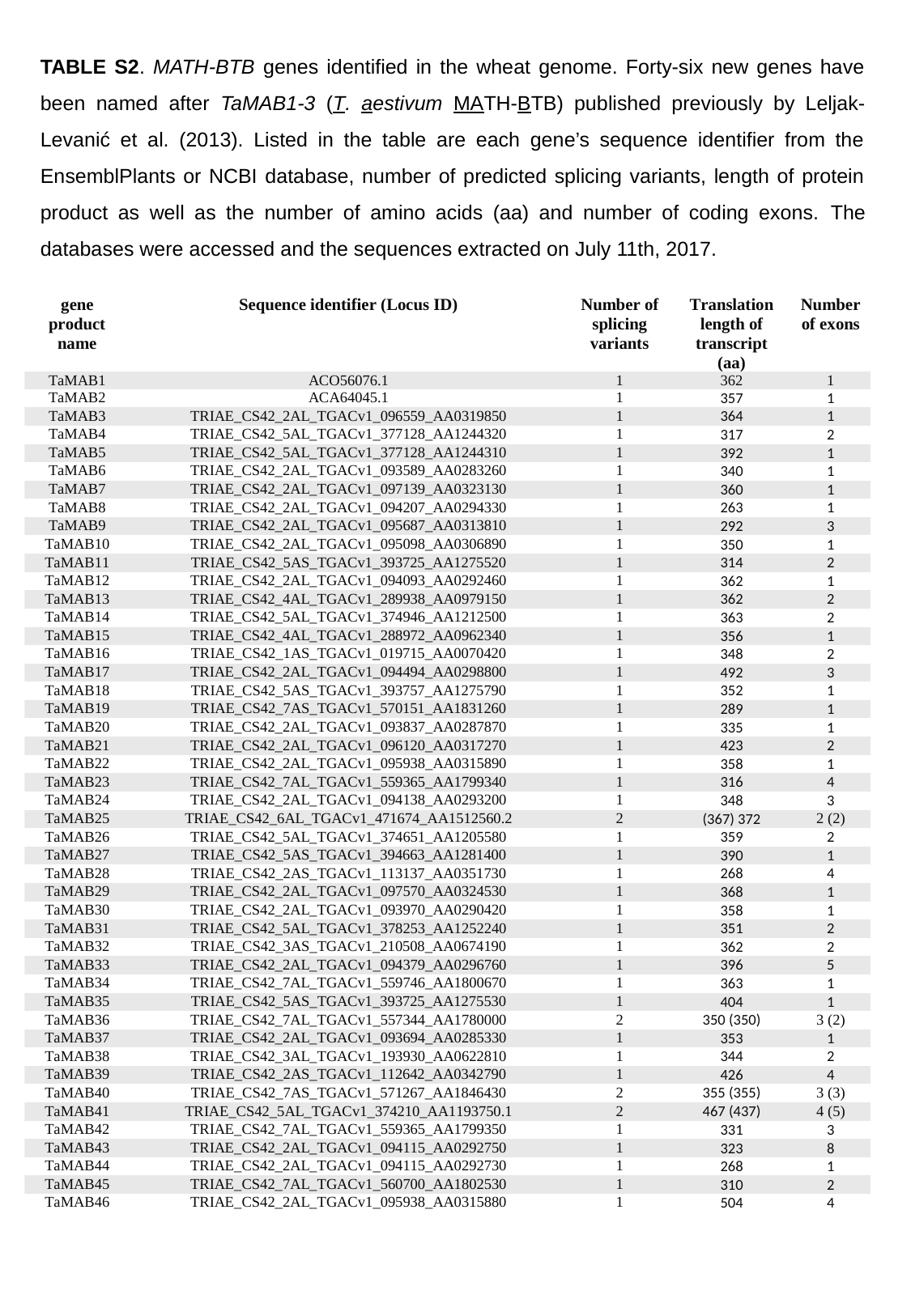

TABLE S2. MATH-BTB genes identified in the wheat genome. Forty-six new genes have been named after TaMAB1-3 (T. aestivum MATH-BTB) published previously by Leljak-Levanić et al. (2013). Listed in the table are each gene’s sequence identifier from the EnsemblPlants or NCBI database, number of predicted splicing variants, length of protein product as well as the number of amino acids (aa) and number of coding exons. The databases were accessed and the sequences extracted on July 11th, 2017.

## Slide 6
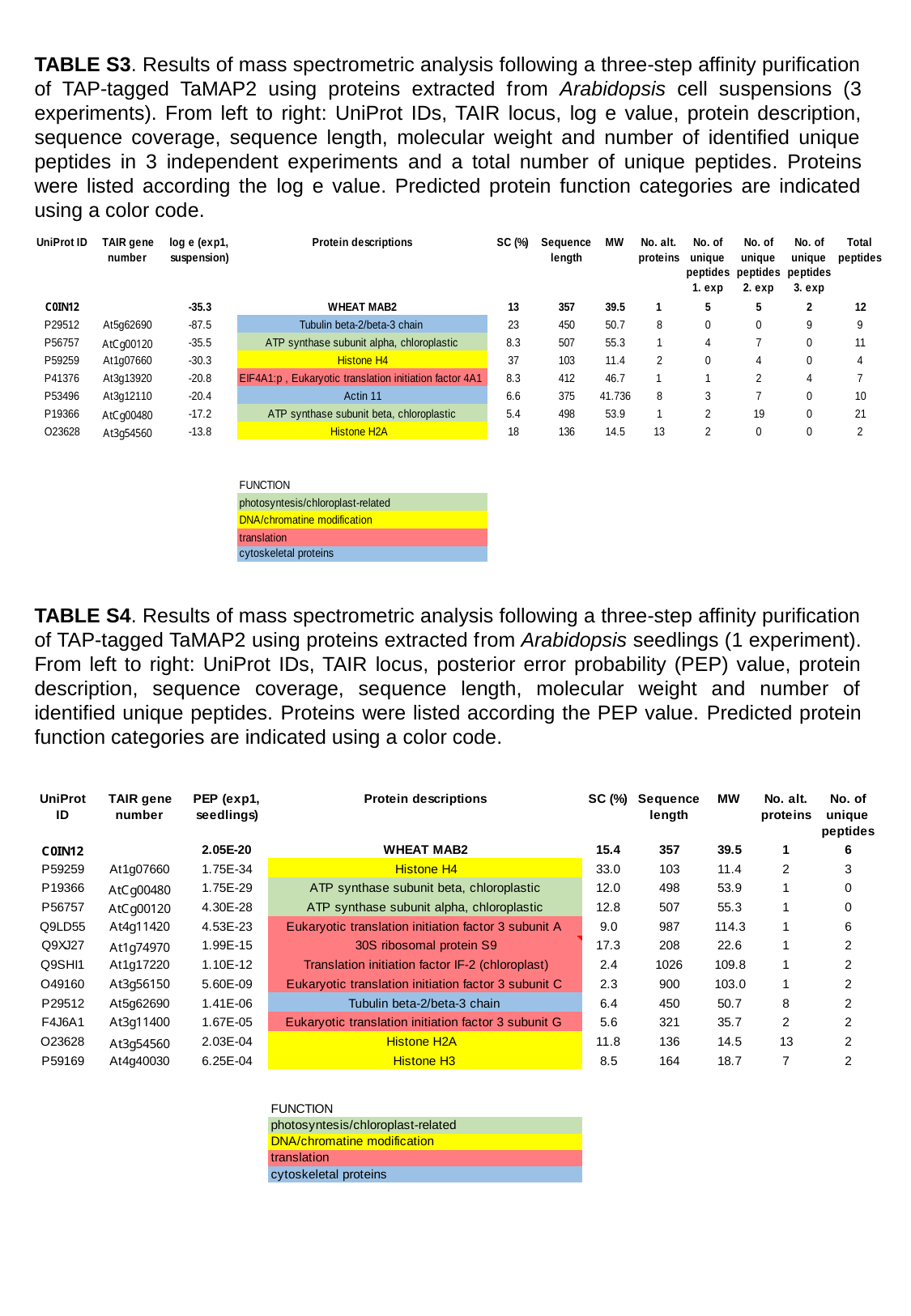

TABLE S3. Results of mass spectrometric analysis following a three-step affinity purification of TAP-tagged TaMAP2 using proteins extracted from Arabidopsis cell suspensions (3 experiments). From left to right: UniProt IDs, TAIR locus, log e value, protein description, sequence coverage, sequence length, molecular weight and number of identified unique peptides in 3 independent experiments and a total number of unique peptides. Proteins were listed according the log e value. Predicted protein function categories are indicated using a color code.
TABLE S4. Results of mass spectrometric analysis following a three-step affinity purification of TAP-tagged TaMAP2 using proteins extracted from Arabidopsis seedlings (1 experiment). From left to right: UniProt IDs, TAIR locus, posterior error probability (PEP) value, protein description, sequence coverage, sequence length, molecular weight and number of identified unique peptides. Proteins were listed according the PEP value. Predicted protein function categories are indicated using a color code.
